# Nearly one in five essential medicines fail quality standards in Africa: A systematic review and meta-analysis of MNCH medicines and antimalarials

**DOI:** 10.1371/journal.pgph.0006326

**Published:** 2026-09-02

**Authors:** Jean Christophe Rusatira, Manuela Dorado Novoa, Jean Berchmans Uwimana, Cameron Persaud, Eishita Pal, Ayesha Khan, Henry Michtalik, Neelaveni Padayachee, Murray Lumpkin, Charles Preston, Saifuddin Ahmed

**Affiliations:** 1 Department of Population, Family and Reproductive Health, Johns Hopkins Bloomberg School of Public Health, Johns Hopkins University, Baltimore, Maryland, United States of America; 2 Department of Pharmacy and Pharmacology, University of the Witwatersrand, Johannesburg, South Africa; 3 Gates Foundation, Seattle, Washington, United States of America; Jhpiego, UNITED STATES OF AMERICA

## Abstract

Substandard and falsified (SF) medicines pose a major threat to maternal, newborn, and child health (MNCH) and malaria control in Africa, yet their true burden remains uncertain. We conducted a systematic review and meta-analysis of studies published between 2010 and 2025 that chemically tested the quality of essential MNCH and antimalarial medicines collected in African countries. Following PRISMA 2020 guidelines and a registered PROSPERO protocol, we searched PubMed, Embase, Web of Science, and WHO databases, screened 3,178 records, and included 123 studies from 34 countries reporting on 36,069 medicine samples. Studies were appraised with the MEDQUARG checklist, and random-effects meta-analyses with Freeman–Tukey transformation were used to estimate pooled SF prevalence overall and by therapeutic class, country, and subregion. Meta-regression explored study-level drivers of heterogeneity. Overall, 17.9% (95% CI 13.6–22.7) of tested medicines failed quality standards. Failure rates were highest for corticosteroids (65.0%), uterotonics (43.9%), and hematinics (34.7%); antimalarials, antihypertensives, and antibiotics ranged from 15-20%. Estimates for corticosteroids and hematinics carried wide confidence intervals, reflecting sparse underlying data. Western and Central Africa had the greatest regional burdens, whereas Southern Africa showed lower pooled estimates but with sparse data. Substandard medicines accounted for most poor-quality products (pooled prevalence 12.8%), while falsified medicines were less common (1.2%). Therapeutic category, geographic subregion, and use of mystery-client purchasing were significant predictors of heterogeneity, whereas publication year and methodological quality were not. Nearly one in five essential MNCH and antimalarial medicines tested in Africa are SF, posing a major barrier to reducing preventable maternal, neonatal, and child mortality. Strengthened regulatory systems, risk-based post-market surveillance, and priority attention to high-risk therapeutics, particularly uterotonics and antimalarials, are urgently needed. As the African Medicines Agency becomes operational, this evidence base offers a concrete foundation for directing continental regulatory investment where it is most needed.

## Introduction

Substandard and falsified (SF) medicines represent a persistent and growing global health threat, with profound implications for Maternal, Newborn, and Child Health (MNCH). The SF medicines compromise treatment effectiveness, prolong illness, increase the risk of antimicrobial resistance, and lead to avoidable morbidity and mortality. According to the 2017 study by the World Health Organization (WHO), approximately 10% of all medicines globally are either substandard or falsified, with the burden disproportionately affecting low- and middle-income countries (LMICs) [1]. The study identified antimalarial medicines, antibiotics and other anti-infective drugs as the most affected drug SF categories. This is particularly concerning in sub-Saharan Africa, where maternal and child mortality rates remain among the highest globally, and malaria and infectious diseases continue to be the leading causes of death in children under five [2,3].

Studies suggest that the prevalence of SF medicines is substantially higher in Africa, compared to Asia and other regions [4]. A study in sub-Saharan Africa found that 14% to 24% of life-saving cancer drugs at health facilities were substandard and falsified [5]. Despite this, comprehensive assessments of SF medicine prevalence across African countries remain limited, underscoring the need to better understand the landscape in this region, particularly for maternal and child health care related medicines.

A striking example is the prevalence of SF oxytocin, a critical drug for managing postpartum hemorrhage, one of the leading causes of maternal death, where between 26.19% and 74.1% of samples in Nigeria were found to be of poor quality, significantly endangering maternal and newborn outcomes [6,7]. The situation is similarly dire for other essential medicines. For instance, in Nigeria, 95% of dexamethasone 4mg/mL ampules were found to be substandard or falsified although the estimate was from a convenience sample, while in Kenya, the figure reached as high as 33% [8,9]. Additional findings include high failure rates for magnesium sulfate (20%) [10], Azithromycin (0–33.3%) [11], oral rehydration salts (76.5%) [12], and gentamicin (71%) [13]. Such widespread medicine quality failures threaten to derail public health gains by rendering key maternal and child interventions ineffective.

The impact of SF antimalarial medicines is particularly devastating. In Nigeria, the prevalence of SF malaria treatments has been estimated at around 20% [6,14–17]. Across Africa, falsified antimalarials are estimated to contribute to between 72,000 and 267,000 deaths of children under five each year [18]. More broadly, SF medicines are responsible for an estimated one million deaths globally every year, including approximately 250,000 child deaths from ineffective treatments for pneumonia and malaria alone [19]. These figures underscore an urgent need to quantify and respond to the burden of SF medicines, not just in terms of prevalence but in their direct impact on health outcomes.

Accurately measuring the burden of SF medicines is especially critical as countries advance their regulatory maturity levels under the WHO Global Benchmarking Tool (GBT) framework. Many countries are at or are transitioning from Maturity Level (ML) 1 or 2, where regulatory systems are present but limited, to Level 3, which indicates a minimally stable, well-functioning regulatory system [20]. Simultaneously, some countries such as Nigeria, Ghana and South Africa are striving to move from Maturity Level 3 to Level 4/ WHO-listed authorities (WLA) for medicines, achieving a regulatory system operating at an advanced level of performance and continuous improvement [20]. Strengthening regulatory systems requires robust evidence to justify investments, guide interventions, and prioritize risk-based surveillance and enforcement strategies. Quantifying the current burden of SF medicines on maternal, newborn, and child mortality, and estimating their impact on disability and premature death they cause, will be instrumental in justifying investments needed to support these countries’ efforts toward higher regulatory maturity.

This meta-analysis is part of the larger “Burden and Effects of Substandard and Falsified Medicines Exposures (BE SAFE)” study, which aims to fill a critical evidence gap by generating robust estimates of SF medicine burden and its health consequences in priority areas such as MNCH. Specifically, this study examines the prevalence of SF maternal, newborn, child health, and antimalarial medicines across African countries to better characterize the scope of the problem in this region. These findings are intended not only to inform national and global health policy responses but also to provide essential data to guide resource allocation, strengthen regulatory systems, and protect vulnerable populations. Importantly, this evidence base is emerging at a pivotal moment, as African countries work toward regulatory strengthening and as the African Medicines Agency (AMA) becomes operational to support regulatory harmonization and coordination across the continent. Quantifying the scale of the problem is therefore essential for supporting regional efforts to enhance regulatory harmonization, coordinate surveillance, and improve the detection of and response to SF medical products across the continent. Results from this study will also contribute to advocacy efforts and help raise awareness of the threats posed by SF medicines.

## Methodology

### Search strategy

This systematic review and meta-analysis followed PRISMA 2020 guidelines [21] and aimed to quantify the prevalence of SF essential medicines used for MNCH [22] and malaria care in Africa. Two distinct search strategies were implemented: one to capture MNCH essential medicines and another focused on antimalarials. Searches were conducted in PubMed, Embase, Web of Science, and World Health Organization (WHO) databases on July 2^nd^, 2025. Reference lists of all included studies were manually reviewed to identify additional eligible publications. The initial search had no geographical limitation and was further restricted to studies conducted on samples from any of the Africa countries.

Search strategies were adapted for each database. For PubMed, MeSH and text-word combinations captured all possible variations of “substandard,” “falsified,” “counterfeit,” “fake,” and “poor-quality” drugs cross-referenced with MNCH- and antimalarial medicine names. The MNCH search included a comprehensive list of WHO-recognized priority medicines such as oxytocin, misoprostol, dexamethasone, magnesium sulfate, tranexamic acid, hematinics, antihypertensives, antibiotics, and oral rehydration salts, among others [22]. The malaria search strategy included antimalarials and artemisinin-based combination therapies (e.g., artemether–lumefantrine, artesunate–amodiaquine, dihydroartemisinin–piperaquine) [18,22]. The comprehensive search strategy and terminology as well as a list of additional sources searched are presented in the supporting information (see S1 Appendix). Full-text records were sought and obtained through the library system of Johns Hopkins University Welch Medical Library. This systematic review and meta-analysis are registered in the PROSPERO database (ID: 1247240) [23].

### Eligibility criteria

Studies were eligible if they reported chemical quality testing of MNCH or antimalarial medicines using pharmacopoeial standards or validated analytical methods, reported prevalence or quantitative active pharmaceutical ingredient (API) content, involved human-use medicines collected from any level of the supply chain, and were published between 2010 and 2025 in English or French. Studies focusing exclusively on veterinary drugs or lacking primary data were excluded. Studies with fewer than 10 medicine samples were excluded from the quantitative synthesis to minimize instability of estimates. This minimum sample-size criterion was applied to the total number of eligible samples contributed by each study, rather than separately to each therapeutic category, country, or other subgroup within a study [24,25].

### Data extraction and definitions

Data were extracted on country of sample collection, year of sample collection, publication year, sampling method, therapeutic category, analytical method, and number of samples classified as substandard or falsified. The specific medicines included within each therapeutic category are listed in S1 Appendix. Following the U.S. Pharmacopeia PQM+ framework, falsified medicines were defined as those with 0% API, an incorrect API, or overt signs of falsification, while substandard medicines were defined as samples containing <90% or >110% of the stated API content, failing dissolution or other pharmacopeial criteria [26,27].

Missing or incompletely reported data were handled using the classifications and level of detail provided by the original investigators. When samples were reported only as “poor quality” without specifying whether they were substandard or falsified, they were classified as substandard unless the investigators explicitly identified them as counterfeit or falsified. When sample-level API content or other testing details were not reported, these values were not imputed, and the samples were classified based on the aggregate results and terminology reported in the original study. Where studies reported only aggregate regional or category-level counts, samples were proportionally allocated across the countries or categories reported in the original study for the relevant subgroup analyses.

Study methodological quality was appraised using the MEDQUARG (Medicine Quality Assessment Reporting Guidelines) checklist [24,25]. Each study was scored on 12 items, including definition of SF medicine, sampling design, analytical method description, validation, and blinding of assessors. Two reviewers independently scored all studies; disagreements were resolved through consensus. Additional information about the MEDQUARG scoring metric used as well as this study’s reported MEDQUARG scores (see S2 Appendix and S3 Appendix).

### Statistical analysis

Prevalence estimates were calculated as the number of SF samples divided by total samples tested. Proportions were transformed using the Freeman–Tukey double-arcsine transformation to stabilize variance [28]. The prevalence statistics were estimated using random-effects meta-analysis with restricted maximum-likelihood (REML) estimation for τ² with inverse-variance weighting to account for between-study heterogeneity. Heterogeneity was quantified using I², τ², and Cochran’s Q statistics [29]. Publication bias was evaluated using a funnel plot analysis with a regression test for funnel plot asymmetry. Baujat and influence plot analyses were conducted to examine which articles contributed the most heterogeneity. Subgroup analyses examined variation by therapeutic category, geographic region, country, and study quality. Mixed and random-effects meta-regression models were used to explore potential confounders, including study methods, therapeutic class, sampling method, publication year, and MEDQUARG score. All analyses were performed in R (v4.3.3) using the *metafor* and *meta* packages.[30]

### Sensitivity analysis

Sensitivity analyses included repeating the analysis using only studies with quality scores ≥6/12 to evaluate whether restricting the sample to higher-quality evidence qualitatively changed the results substantially. The influence of individual studies was also assessed using leave-one-out procedures, Baujat plot test, Cook’s distance, and DFBETAS diagnostics. Publication bias was assessed using funnel plots and Egger’s regression test [31,32].

## Results

### Literature search

**Fig 1:**
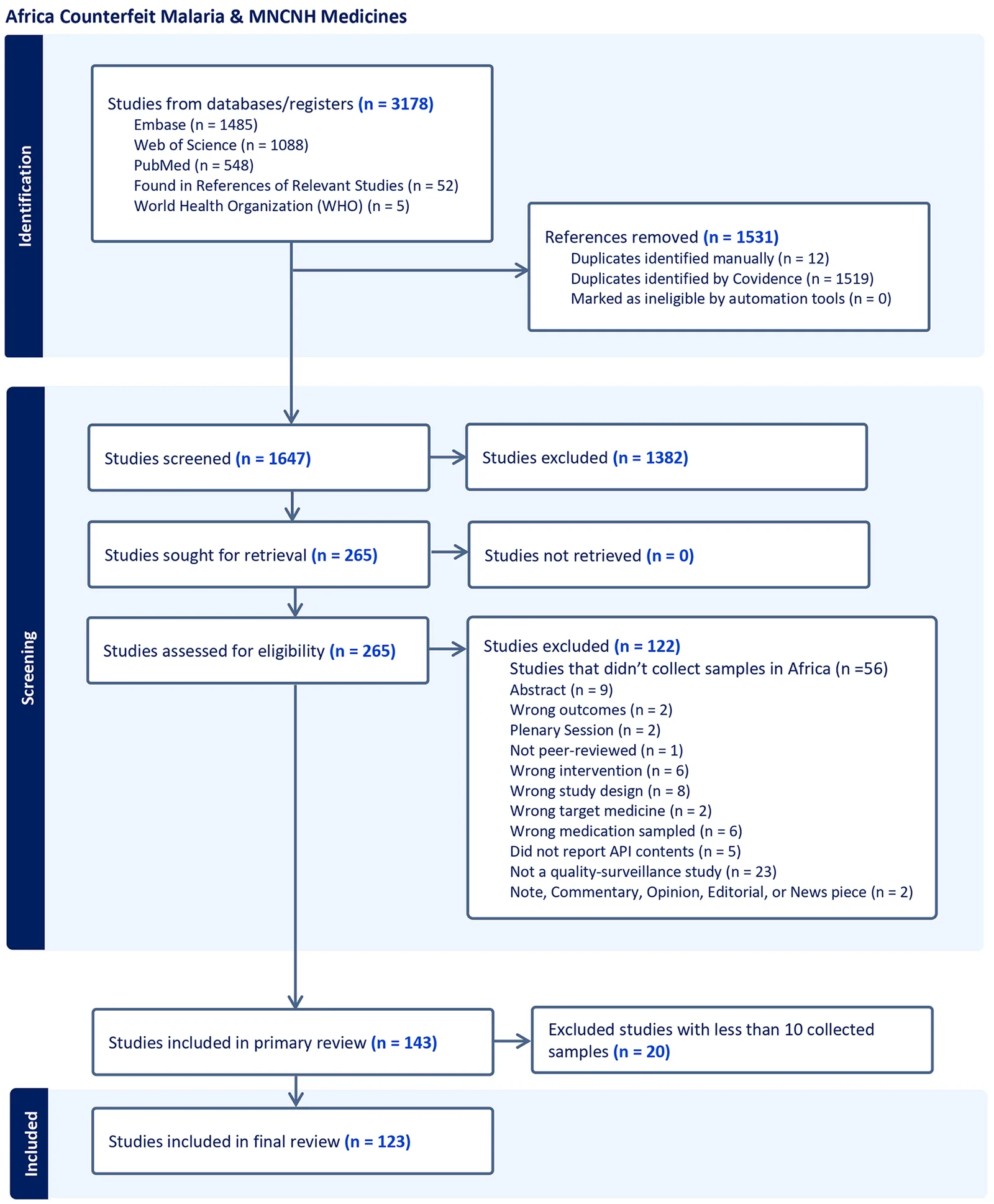
PRISMA flowchart.

**Fig 2:**
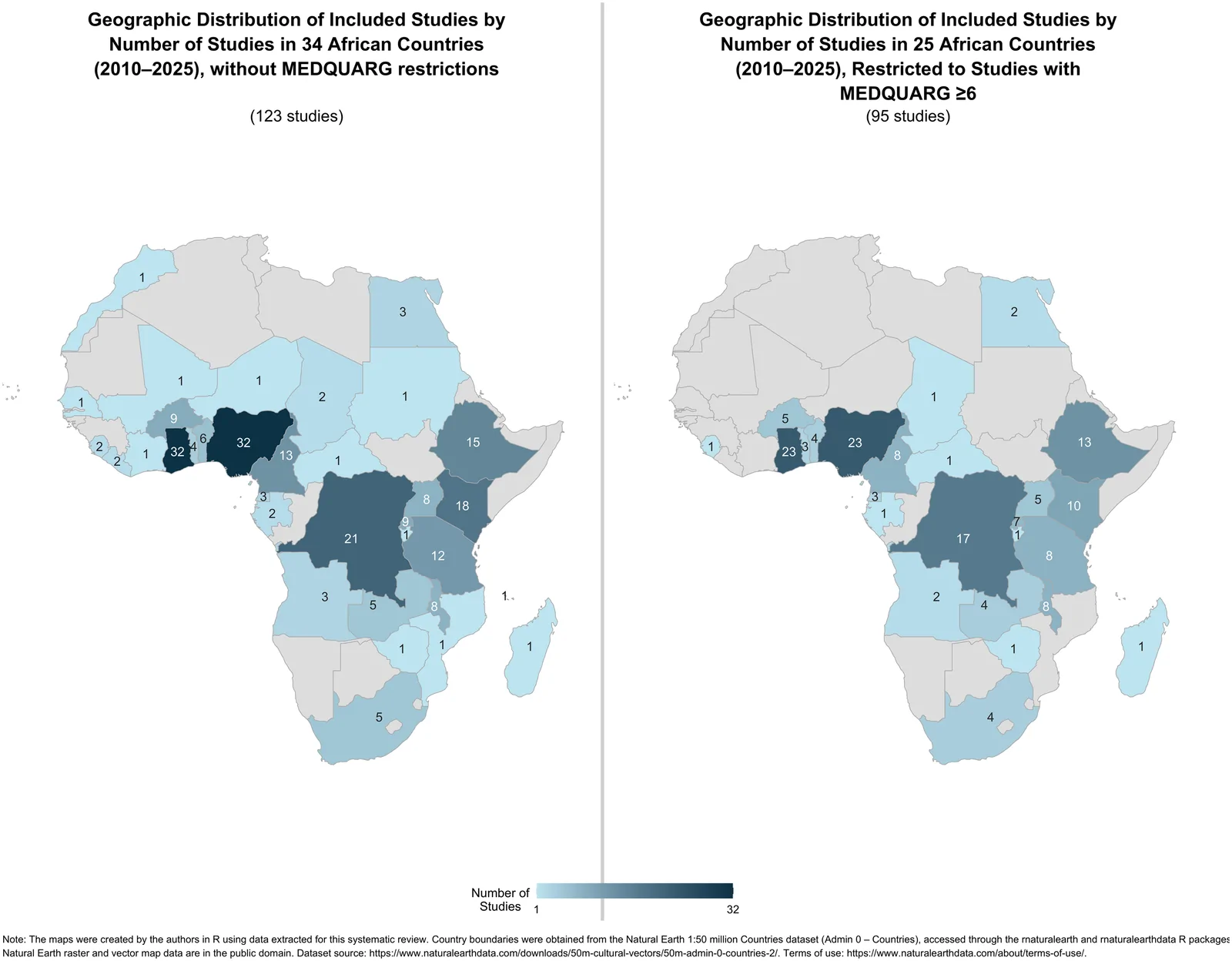
Distribution of medicine quality studies across Africa (2010–2025) with and without study quality restrictions.

The initial search had no geographical limitation and returned 3,178 citations from databases, references from relevant studies, and WHO reports. (Fig 1) After removing duplicates and screening, a total of 143 studies that included one or more African countries were eligible for inclusion based on PRISMA criteria, all of which involved collection of essential maternal, child health, or antimalarial medicines and reporting of substandard and/or falsified medicines. To ensure reliability in effect estimates, studies with fewer than 10 samples were excluded, yielding 123 studies across 34 countries and comprising 36,069 medicine samples. A complete list of the 123 studies included in the meta-analysis is provided in S4 Appendix. Table 1 summarizes the therapeutic categories included in the meta-analysis and presents, for each category, the countries represented, number of medicine samples, number of contributing studies, and pooled prevalence estimates. In addition, Fig 2 presents a heatmap showing the geographic distribution of included studies across Africa, and S3 Appendix presents the MEDQUARG scores for all included studies. S10 Appendix lists the studies excluded from this meta-analysis because they included fewer than 10 samples.

**Table 1:** List of therapeutic categories included in the meta-analysis.

Therapeutic Category	Country	# Samples	# Studies	Random Effects Prevalence (95% CI)
Analgesic	Burundi, Cameroon, Central African Republic, Chad, DR Congo, Ethiopia, Ghana, Kenya, Malawi, Nigeria, Rwanda, South Africa, Tanzania, Uganda	820	15 studies	3.9% (0.8%–8.6%)
Antibiotic	Benin, Burkina Faso, Burundi, Cameroon, Central African Republic, Chad, DR Congo, Ethiopia, Ghana, Kenya, Liberia, Madagascar, Malawi, Mozambique, Nigeria, Rwanda, Sierra Leone, South Africa, Tanzania, Togo, Uganda, Zimbabwe	3217	45 studies	15.7% (9.0%–23.6%)
Anticonvulsant	Burkina Faso, Kenya, Nigeria, Tanzania, Uganda, Zimbabwe	264	3 studies	0.2% (0.0%–2.9%)
Antidiarrheal	Kenya, Nigeria, Tanzania, Uganda, Zimbabwe	18	2 studies	0.0% (0.0%–8.8%)
Antifibrinolytic	Kenya, Nigeria, South Africa, Tanzania	14	1 study	0.0% (0.0%–11.9%)
Antihypertensive	Burundi, Cameroon, Central African Republic, Chad, DR Congo, Kenya, Malawi, Nigeria, Uganda	166	4 studies	18.9% (0.0%–59.3%)
Antimalarial	Angola, Benin, Burkina Faso, Burundi, Cameroon, Central African Republic, Chad, Comoros, DR Congo, Egypt, Equatorial Guinea, Ethiopia, Gabon, Ghana, Ivory Coast, Kenya, Liberia, Malawi, Mali, Morocco, Mozambique, Niger, Nigeria, Rwanda, Senegal, Sierra Leone, Tanzania, Togo, Uganda, Zambia	26810	68 studies	19.4% (13.4%–26.1%)
Antiretroviral	Burkina Faso, Cameroon, DR Congo, Ethiopia, Kenya, Nigeria, Rwanda, South Africa, Tanzania, Zambia	3293	8 studies	0.5% (0.3%–0.8%)
Contraceptive	Kenya, Madagascar, Tanzania, Uganda, Zimbabwe	12	2 studies	0.0% (0.0%–13.9%)
Corticosteroid	Kenya, Madagascar, Nigeria, Tanzania, Uganda, Zimbabwe	32	2 studies	65.0% (6.2%–100.0%)
Electrolyte	Kenya, Nigeria	153	3 studies	0.0% (0.0%–1.7%)
Hematinic	Ghana, Sudan	39	3 studies	34.7% (0.0%–85.5%)
Uterotonic	Burkina Faso, DR Congo, Egypt, Ethiopia, Ghana, Kenya, Madagascar, Malawi, Nigeria, Rwanda, South Africa, Tanzania, Uganda, Zimbabwe	1229	14 studies	43.9% (24.7%–63.9%)

### Pooled prevalence

**Fig 3:**
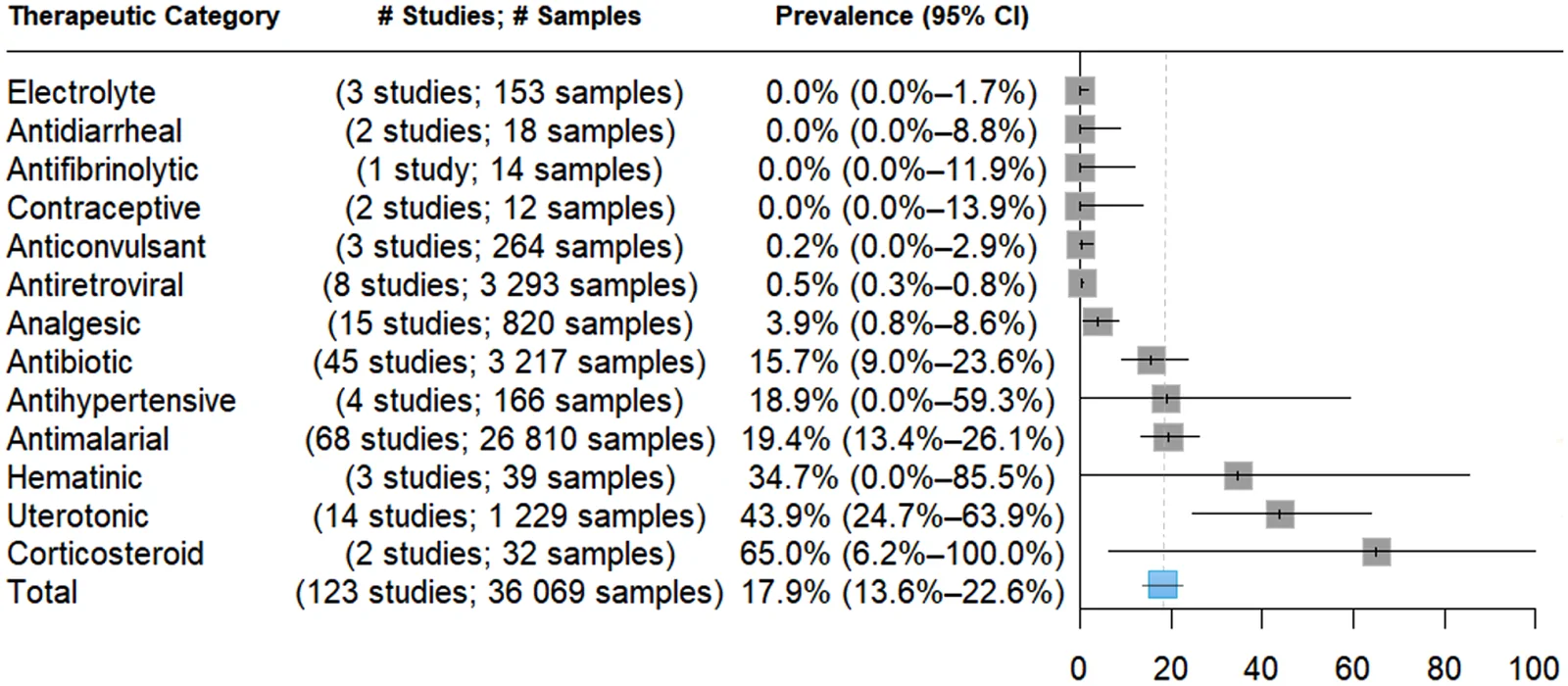
Pooled prevalence of substandard and falsified essential MNCH and antimalarial medicines across 34 African countries (2010–2025), by therapeutic category. Note: Individual studies may contribute data to more than one therapeutic category. As a result, the sum of studies across categories exceeds the total number of unique studies included in the meta-analysis (N = 123). The “Total” row reflects unique studies and samples counted once.

The results from random-effects meta-analysis model using a Freeman–Tukey double-arcsine transformation revealed the pooled prevalence of SF medicines of 17.9% (95% CI 13.6–22.7; see S5 Appendix). Between-study heterogeneity was high across categories (I² = 97.8%; τ² = 0.0961; Q = 5671.83, p < 0.001). Among core MNCH medicines, corticosteroids showed the highest prevalence of SF medicines at 65% (95% CI 6.2–100; 2 studies), uterotonics 43.9% (95% CI 24.7–63.9; 14 studies) and hematinics 34.7% (95% CI 0.0–85.5, 3 studies) (Fig 3). Antimalarials had a pooled prevalence of 19.4% (95% CI 13.4–26; 68 studies), antihypertensives showed a prevalence of 18.9% (95% CI 0.0–59.3; 4 studies), and antibiotics 15.7% (95% CI 9.0–23.6; 45 studies).

### Geographical differences in prevalence

**Fig 4:**
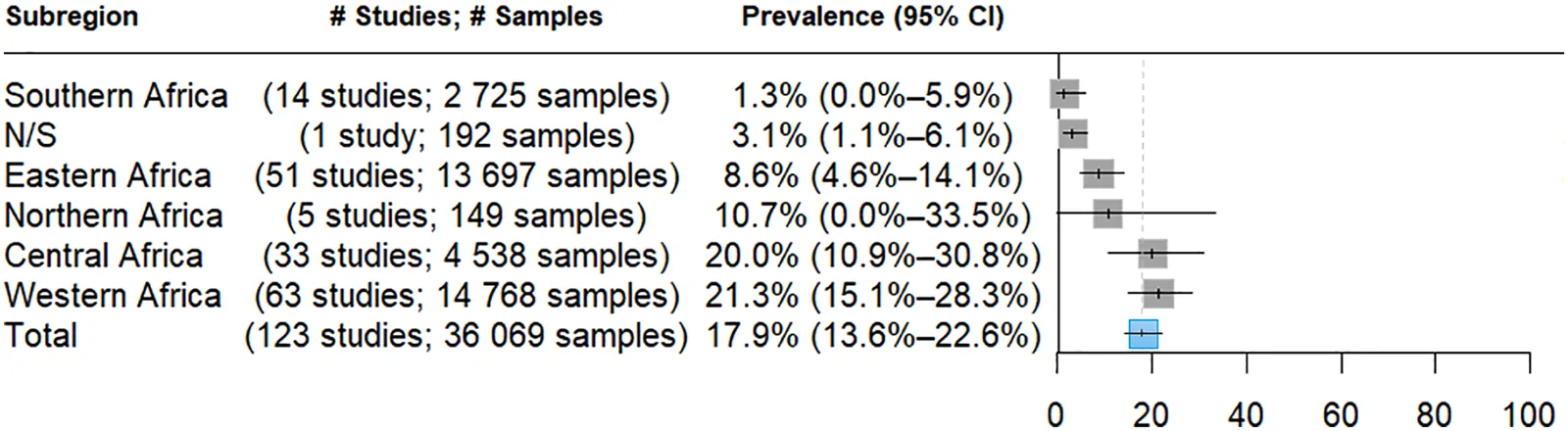
Regional variation in pooled prevalence of substandard and falsified essential MNCH and antimalarial medicines in Africa (2010–2025). *Note: N/S was included due to the study Bernier et al., 2016* [39] *which reported having collected samples from sub-Saharan Africa, however there was no specification as to which countries.*

To assess whether the substantial heterogeneity observed in the overall model reflected systematic differences across study characteristics, we conducted subgroup meta-analyses by geographic region and country. Among countries with sufficiently large samples of studies (at least three studies), prevalence estimates of substandard or falsified MNCH and antimalarial medicines varied substantially (see S5 Appendix). The highest pooled prevalence was observed in the Democratic Republic of Congo (29.3%, 95% CI: 15.1–45.5; 21 studies), followed by Ghana (23.6%, 95% CI: 13.3–35.4; 32 studies), Nigeria (21.0%, 95% CI: 13.1–30.1; 32 studies). In contrast, countries with similarly substantial evidence bases but lower pooled estimates included Ethiopia (5.5%, 95% CI: 1.4–11.5; 15 studies), Tanzania (5.2%, 95% CI: 0.4–13.2; 12 studies), and Rwanda (5.7%, 95% CI: 0.8–13.8; 9 studies). The highest regional prevalence estimates of SF MNCH and antimalarial medicines were observed in Western Africa (21.3%, 95% CI: 15.1–28.3) and Central Africa (20.0%, 95% CI: 10.9–30.8) (Fig 4). Northern Africa showed a moderate prevalence of 10.7% (95% CI: 0.0–33.5), followed by Eastern Africa (8.6%, 95% CI: 4.6–14.1). The lowest pooled prevalence was observed in Southern Africa (1.3%, 95% CI: 0.0–5.9). For reference, S6 Appendix presents a list of Africa showing country-level regional classifications used in this analysis.

**Fig 5:**
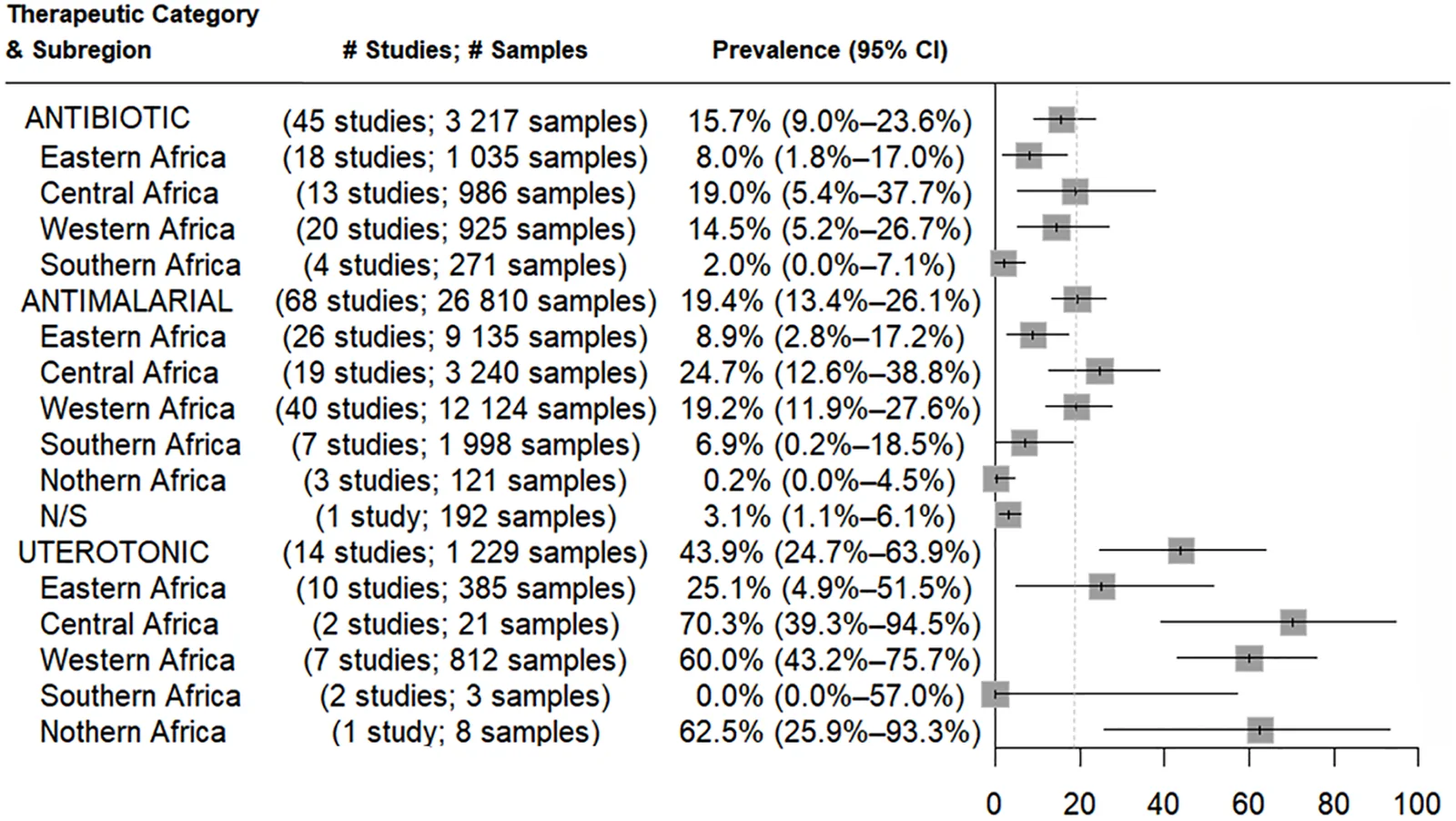
Regional variation in pooled prevalence of substandard and falsified essential MNCH and antimalarial medicines in Africa (2010–2025) by therapeutic category, Without MEDQUARG restrictions.

When subregional patterns were examined within each therapeutic category (Fig 5), additional heterogeneity emerged beyond the overall regional and category-level estimates previously described. Uterotonic medicines exhibited the highest and most variable SF prevalence across subregions. The highest prevalence estimates were 70.3% (95% CI: 39.3–94.5), 62.5% (95% CI: 25.9–93.3) and 60.0% (95% CI: 43.2–75.7) in Central Africa, Northern Africa and Western Africa, respectively. Eastern Africa showed a prevalence of 25.1% (95% CI: 4.9–51.5). The sample size for Southern Africa was very small, hence no reliable estimates could be estimated. For antimalarial medicines, Central Africa and Western Africa showed the highest prevalence estimates (24.7%, 95% CI: 12.6–38.8, and 19.2%. 95% CI: 11.9–27.6, respectively). For antibiotics, prevalence estimates varied markedly by subregion, ranging from 2.0% (95% CI: 0.0–7.1) in Southern Africa to 19.0% (95% CI: 5.4–37.7) in Central Africa. Prevalence estimates were 8.0% (95% CI: 1.8–17.0) in East Africa and 14.5% (95% CI 5.2–26.7) in Western Africa.

### Determinants of heterogeneity from meta-regression model

Mixed-effects meta-regression identified therapeutic category, subregion, and mystery-client purchasing as statistically significant study-level factors associated with variation in SF prevalence. Therapeutic category demonstrated a highly significant omnibus effect (QM = 83.75; p < 0.0001), indicating substantial differences in prevalence across medicine classes. Subregion was also statistically significant (QM = 29.36; p < 0.0001), reflecting marked geographic variation in SF prevalence across African regions. The mystery-client approach showed a statistically significant association with higher SF prevalence (QM = 4.76; p = 0.03). In contrast, publication year (p = 0.29), number of samples tested (p = 0.07), MEDQUARG score modeled continuously (p = 0.73) or as a binary threshold (≥6; p = 0.78), and use of random sampling (p = 0.64) were not statistically significant and did not meaningfully reduce τ².

### Sensitivity and publication bias diagnostics

**Fig 6:**
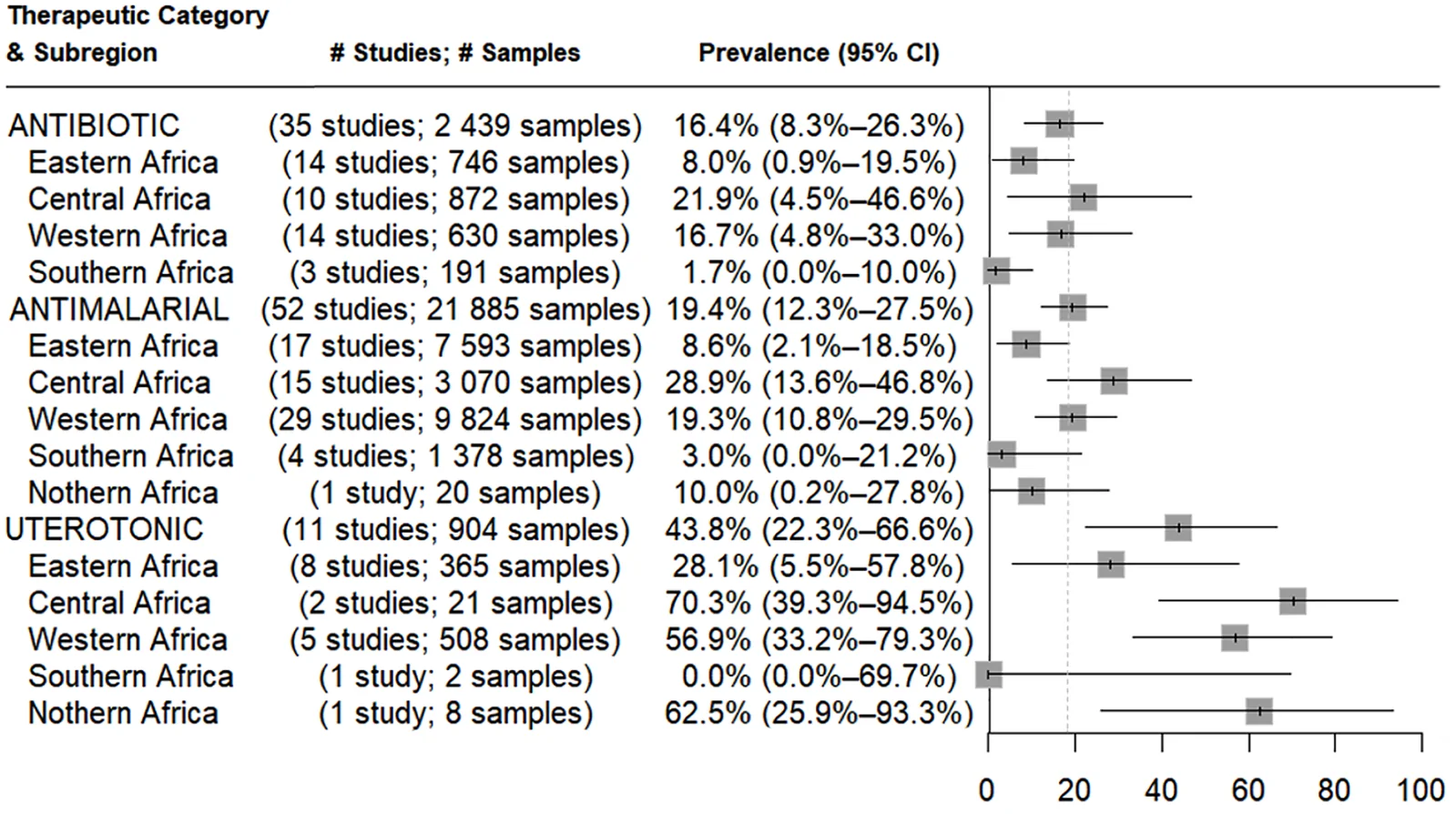
Regional variation in pooled prevalence of substandard and falsified essential MNCNH and antimalarial medicines in Africa (2010–2025) by therapeutic category, Restricted to studies with MEDQUARG ≥6.

Restricting the dataset to higher-quality studies (MEDQUARG ≥ 6) did not significantly reduce the pooled prevalence, which remained at 17.9% (95% CI: 13.6%–22.6%) across all 123 studies with no MEDQUARG restrictions, and 18.2% (95% CI: 13.1%–24.0%) among all 95 higher-quality studies (after MEDQUARG ≥ 6 restrictions; S7 Appendix). However, several country-level variations emerged (Fig 6). For example, uterotonic medicines continued to show the highest prevalence across subregions, although estimates were highly variable. Prevalence estimates were higher in Central Africa (70.3%, 95% CI: 39.3–94.5), Western Africa (56.9%, 95% CI: 33.2–79.3), and Northern Africa (62.5%, 95% CI: 25.9–93.3). Eastern Africa showed a prevalence of 28.1% (95% CI: 5.5–57.8).

For antimalarial medicines, prevalence was highest in Central Africa (28.9%, 95% CI: 13.6–46.8), followed by Western Africa (19.3%, 95% CI: 10.8–29.5). Lower prevalence was observed in Eastern Africa (8.6%, 95% CI: 2.1–18.5) and Southern Africa (3.0%, 95% CI: 0.0–21.2), while estimates for Northern Africa (10.0%, 95% CI: 0.2–27.8) were based on very limited data.

For antibiotics, prevalence estimates showed similar patterns across subregions, with higher prevalence in Central Africa (21.9%, 95% CI: 4.5–46.6), Western Africa (16.7%, 95% CI: 4.8–33.0) and Eastern Africa (8.0%, 95% CI: 0.9–19.5) compared to Southern Africa (1.7%, 95% CI: 0.0–10.0).

At the therapeutic-category level, no changes were statistically significantly different after restricting the analysis to higher-quality studies. The uterotonics showed a similar pooled prevalence (43.8%, 95% CI: 22.3–66.6; 11 studies) compared to the unrestricted estimate of 43.9% (95% CI: 24.7–63.9; 14 studies). Similarly, across regions, prevalence estimates for the different therapeutic categories remained similar.

In Western Africa, the pooled prevalence of SF uterotonics changed slightly from 60.0% (95% CI: 43.2–75.7; 7 studies) to 56.9% (95% CI: 33.2–79.3; 5 studies) and the prevalence of SF antibiotic changed from 14.5% (95% CI: 5.2–26.7; 20 studies) to 16.7% (95% CI: 4.8–33.0; 14 studies). In Central Africa, the prevalence estimates for antibiotics changed from 19.0% (95% CI: 5.4–37.7; 13 studies) to 21.9% (95% CI: 4.5–46.6; 10 studies), while for antimalarial medicines, estimates changed from 24.7% (95% CI: 12.6–38.8; 19 studies) to 28.9% (95% CI: 13.6–46.8; 15 studies).

Similarly, the prevalence estimates for Antimalarial and antibiotics were consistent for Eastern Africa, while uterotonic prevalence in this region changed from 25.1% (95% CI: 4.9–51.5; 10 studies) to 28.1% (95% CI: 5.5–57.8; 8 studies). In Northern Africa, except for antimalarial prevalence that did not have sufficient sample size, other prevalence estimates for the other categories remained the same.

Further, when prevalence estimates were disaggregated by whether the medicine was falsified or substandard, clear differences emerged between falsified and substandard products at the continental level (see S8 Appendix). Across all included studies prior to MEDQUARG restriction (123 studies), the pooled prevalence of falsified medicines was 1.2% (95% CI: 0.5–2.1), whereas substandard medicines accounted for the majority of poor-quality products, with a pooled prevalence of 12.8% (95% CI: 8.9–17.2). After restricting analyses to higher-quality studies (MEDQUARG ≥6; 95 studies), the prevalence estimates remained similar. The pooled prevalence of falsified medicines was 1.1% (95% CI: 0.3–2.2), while the prevalence of substandard medicines was 13.2% (95% CI: 8.7–18.4). An important note to make is that, the subgroup-specific prevalence estimates do not sum to the overall pooled SF prevalence (17.9%) because falsified, substandard, and combined SF estimates were generated using separate random-effects meta-analyses with independently estimated between-study heterogeneity, which differed across models (τ² = 0.0223 for falsified, 0.1051 for substandard, and 0.0961 for combined SF).

Lastly, the influence diagnostics identified five studies, Gabel et al., 2023 [41]; Chikowe et al., 2015 [40]; Wynendaele et al., 2019 [49]; Stanton et al., 2012 [47]; and Bekoe et al., 2020 [38], as contributing the most to heterogeneity, but sequentially removing each produced negligible changes in τ² or the pooled prevalence estimate, indicating that no single study disproportionately influenced the results. Evidence of publication bias was observed. The funnel plot demonstrated visible asymmetry, with smaller studies tending to report higher prevalence estimates, and Egger’s regression test confirmed statistically significant small-study effects (t = 3.78, df = 121, p = 0.0002). (see S9 Appendix)

## Discussion

This systematic review and meta-analysis, drawing on more than 36,000 medicine samples across 123 studies from 34 African countries, provides one of the most comprehensive assessments to date of SF essential MNCH medicines and antimalarials on the continent. A first takeaway is that there are many countries across Africa with no or few SF studies, suggesting that our knowledge of the situation is limited, and may be an underestimate of the problem. From the available studies, the pooled prevalence of 18% SF observed here is nearly double the widely cited 10% estimate for LMICs from the WHO’s 2017 impact study, [1] underscoring persistent medicine-quality failures in high-burden clinical areas. Beyond antimalarials, historically the best-studied class, we find substantial quality failures in uterotonics, antihypertensives, corticosteroids, hematinics, and antibiotics which are critical MNCH medicines whose continental burden had not previously been systematically synthesized [4,7–13,23]. However, several therapeutic-category estimates were based on only one to three studies and fewer than 40 samples, including contraceptives, antifibrinolytics, antidiarrheals, corticosteroids, and hematinics. These estimates have limited precision and generalizability and should therefore be interpreted cautiously.

These medicines are central to preventing postpartum hemorrhage, managing hypertensive disorders of pregnancy, improving preterm neonatal outcomes, and preventing neural tube defects and maternal anemia [2–6]. In sub-Saharan Africa, where postpartum hemorrhage remains the leading cause of maternal deaths and poor-quality oxytocin and misoprostol have been repeatedly documented in Nigeria, Kenya, Ghana, and DRC [6–9], the high prevalence of SF uterotonics likely contribute directly to preventable maternal mortality while failures in corticosteroid quality threaten neonatal survival among preterm infants. These findings collectively indicate that medicine quality is a major and under-recognized barrier to achieving regional targets for maternal and neonatal mortality reduction.

Antimalarials showed a substantial pooled SF prevalence of around 19%, consistent with long-standing concerns regarding poor-quality Artemisinin-based combination therapies (ACTs) in malaria-endemic settings [6,14–17]. Because malaria remains a leading cause of under-five mortality in Africa, failures in ACT quality threaten treatment efficacy, facilitate resistance, and may contribute to the tens of thousands of estimated child deaths annually attributable to SF antimalarials [18,20]. The persistence of poor-quality antimalarials over more than a decade, despite multiple donor-supported surveillance programs and national ACT quality initiatives, underscores structural vulnerabilities in supply-chain oversight, market authorization, procurement, and wholesale distribution. These vulnerabilities are compounded in West and Central Africa by porous borders, informal drug markets, limited cold-chain infrastructure, and fragmented wholesaler networks that facilitate circulation of degraded or falsified products [6,7,18].

We also observed marked regional and country-level heterogeneity, with consistently higher SF prevalence in West and Central Africa and substantially lower in Southern Africa and parts of Northern African. These patterns persisted even after restricting analyses to studies with higher methodological quality (MEDQUARG ≥6), suggesting that the differences may reflect genuine disparities in regulatory and supply-chain performance but in Southern and Eastern Africa the evidence base is sparse, sample sizes are modest, and sampling frames often exclude informal markets, so lower pooled estimates likely understate the true burden. In Southern Africa, low estimates likely reflect sampling bias toward formal, donor-upported supply chains where SRA-approved products predominate, with informal markets largely excluded. In Eastern Africa, screening tools with limited sensitivity further constrain detection; Gnegel et al., 2022 [42] cautioned that the GPHF Minilab likely missed a considerable number of substandard samples.

Methodological features of the underlying studies further shape the observed variation. Many studies relied primarily on screening tools such as Minilab thin-layer chromatography, visual inspection, or handheld spectrometry, applying relatively permissive API thresholds and rarely confirming results with compendial testing (e.g., HPLC, dissolution, impurity profiling). For instance, in several South African studies, failures identified through visual inspection, labeling irregularities, inappropriate repackaging, and storage practices were not incorporated into the quantitative SF prevalence used for meta-analysis, despite representing clinically meaningful quality defects. In Lehmann et al., 2018 [44], for example, nearly half of samples failed at least one quality criterion, predominantly due to packaging and handling deficiencies, yet only a small fraction failed API-based assays—suggesting that chemical testing alone likely underestimates the true burden of poor-quality medicines.

Definitions of SF medicines varied with some studies limited failure to assay outside the study-specific pharmacopoeial range, whereas others included dissolution, sterility, microbial contamination, or physical defects, complicating direct comparison. Large antimalarial studies further demonstrate the sensitivity of reported SF prevalence to analytical thresholds. For instance, the ACT Consortium, 2015 [34] used a broad API acceptance range of 85–115%, reporting 4.1% substandard artemisinin derivatives and 12.1% substandard ACTs. Narrowing the range to 90–110% raised these figures to 17.6% and 30.6% respectively—showing that low prevalence estimates can reflect permissive cutoffs rather than true product quality. Similarly, Ahmed et al., 2024 [35] reported 58.3% substandard antimalarials based on failure of any quality parameters, while API-only reclassification yielded markedly lower estimates, underscoring how SF prevalence shifts depending on reporting conventions. To harmonize comparisons, we applied a uniform 90–110% threshold, reclassifying samples assessed against broader ranges as substandard—consistently revealing higher SF prevalence than originally reported. Where studies reported only regional or category-level counts, samples were proportionally imputed across listed countries, so 0% country-level estimates should be read as reflecting reporting granularity rather than confirmed absence [36,37,43]. The markedly lower pooled prevalence of falsified products compared with substandard products should also be interpreted cautiously. Many studies focused primarily on API content or other pharmacopeial quality parameters and did not consistently distinguish falsified products from substandard products. In addition, samples described only as “poor quality” were classified as substandard unless explicitly reported as counterfeit or falsified. In addition, confirmed falsified products are often intercepted by law enforcement and customs, and may not reach the sampling frames of the studies pooled here.The pooled falsified estimate may therefore be conservative and should not be interpreted as evidence that falsified products are absent or necessarily uncommon.

Beyond API content, several investigations identified microbiological and physical specifications such as contamination, inadequate hardness, friability, weight variation, and labeling or packaging deficiencies that pose risks even when chemical content is acceptable [33,45]. These dimensions were not routinely incorporated into SF prevalence metrics, contributing to underestimation of clinically important quality problems. Financial constraints were repeatedly cited as barriers to full compendial testing; Petersen et al., 2017 [46] noted that confirmatory testing for 900 samples would have cost $390,000, a tenfold increase over screening costs.

Meta-regression analysis identified therapeutic category, geographic subregion, and mystery-client purchasing as significant sources of heterogeneity, whereas publication year and methodological quality were not significantly associated with SF prevalence. The higher prevalence observed in studies using mystery-client purchasing may reflect the ability of this approach to capture products as they are ordinarily sold because vendors are unaware that they are being evaluated and therefore cannot selectively provide higher-quality products. Mystery-client methods may also be used more frequently in informal or suspected high-risk outlets. This association should not be interpreted as causal, as it may partly reflect differences in outlet type, sampling strategy, and market setting. The absence of a significant association with publication year suggests that medicine-quality problems persisted throughout the study period.

These findings have direct implications for the operationalization of the AMA and for countries advancing through WHO GBT maturity levels [18,20]. A continental understanding of where SF medicines are most concentrated and in which therapeutic areas can guide risk-based prioritization, regulatory investment, and coordinated regional action. Given the high failure rates observed for uterotonics, antihypertensives, and antimalarials [6–9], these products should be early candidates for post-market surveillance and compendial testing and analytics capacity strengthening at the national, regional, and continental levels, particularly in settings with limited regulatory capacity. Prioritizing these high-risk classes aligns with country and AMA efforts to progress from ML2 to ML3 and ML4/WLA and to establish more stable, well-functioning regulatory systems under the WHO GBT framework [20]. These findings also indicate that as countries progress towards ML4/WLA, investments will be needed to implement nationally representative sampling frames that include informal and private markets, harmonized SF definitions, and systematic use of pharmacopeial methods alongside rapid screening tools.

Achieving this will also require interoperable regional data-sharing systems capable of detecting cross-border circulation of SF products [18], regional reference laboratories to support countries lacking advanced analytical capacity [26,27] and hybrid surveillance models that combine random, risk-based, and sentinel sampling to capture both routine and high-risk signals [24,25,43]. In addition, digital and pharmacovigilance-linked early-warning mechanisms, drawing from community treatment-failure reports, facility-level feedback, and packaging-anomaly indicators, can enhance detection of emerging quality threats. These systems can also support the identification of manufacturers or suppliers associated with recurrent quality failures, enabling the development of national and continental watchlists and more targeted regulatory follow-up. These approaches mirror regulatory innovations promoted by WHO, Africa CDC, and other global health partners and are essential for accelerating NRA’s transition from ML2 to ML3 transitions, especially in countries preparing for AMA treaty ratification [18,20].

Funnel-plot asymmetry and Egger’s regression test indicated small-study effects [31,32]. These findings may reflect publication bias, selective reporting, or systematic differences between smaller and larger studies in therapeutic category, study setting, and methodological characteristics; however, the available data do not support attributing them primarily to risk-based sampling. Estimates based on sparse evidence should therefore be interpreted cautiously. The corticosteroid estimate was based on only two studies and 32 samples, with study-specific failure proportions of 20/22 and 3/10, respectively [8,48]. Its wide confidence interval indicates substantial imprecision, and the estimate should not be interpreted as representative of corticosteroid quality across Africa. Sensitivity analyses, including leave-one-out diagnostics and restriction to studies with MEDQUARG scores ≥6, showed that no single study materially influenced the overall pooled estimate (see S7 Appendix and S9 Appendix).

Despite the limitations mentioned above, this review benefits from several strengths: the largest dataset assembled to date on MNCH and antimalarial medicine quality in Africa [1,6–19], comprehensive and multilingual searches [21,22], adherence to PRISMA 2020 guidelines [21], rigorous quality assessment using MEDQUARG [24,25], and extensive subgroup, sensitivity, and meta-regression analyses.

## Conclusion

This meta-analysis shows that SF MNCH and antimalarial medicines pose a major under-recognized public health threat across Africa, with nearly 17.9% of tested products failing quality standards. This statistic is nearly double the widely cited 10% estimate for LMICs. Although the available literature remains limited, with many countries still lacking quality surveillance data, medicine-quality failures remain a systemic barrier to achieving regional and global health targets for women and children, particularly given the high failure rates observed in uterotonics, antihypertensives, corticosteroids.

The pronounced geographic disparities observed across countries and regions, persistent even when analyses were restricted to higher-quality studies, suggest structural differences in regulatory capacity, supply-chain management, and post-market surveillance rather than random variation. Addressing these gaps will require strengthening routine and risk-based sampling, expanding laboratory capacity, and improving data systems that enable regulators to detect and respond to quality defects more rapidly. As the African Medicines Agency becomes operational, this continental evidence base offers a concrete foundation for prioritizing high-risk therapeutic classes and directing regulatory investment where it is most urgently needed.

Future research should prioritize nationally representative sampling, standardized SF definitions, and linkage of surveillance findings with clinical outcomes to quantify the true health burden of poor-quality medicines. Economic evaluations estimating the costs of treatment failure, antimicrobial resistance, and avoidable deaths will strengthen the case for regulatory investment, while evaluations of WHO GBT maturity-level transitions and AMA-supported reforms, will be essential for identifying which interventions most effectively reduce these burdens. Ensuring equitable access to safe, effective, and quality-assured MNCH and antimalarial medicines remains critical to safeguarding public health, strengthening health system resilience, and sustaining gains in maternal, newborn, and child survival across the continent.

### Use of AI tools

ChatGPT (OpenAI) and Grammarly were used solely to support grammatical editing, wording clarity, and orthographic consistency. All scientific content, analyses, and interpretations were conducted by the authors.

### Data sharing

All data underlying this systematic review and meta-analysis are available within the manuscript and its supplementary appendices, including the full search strategies (S1 Appendix), MEDQUARG quality assessment criteria and scores (S2 and S3 Appendices), study-level prevalence estimates (S5 Appendix), and complete analysis dataset (S11 Appendix). All data were extracted from the included studies and their supplementary materials; no confidential or restricted-access data were used.

## Supporting information

S1 Appendix Search strategy. Full search strategies used to identify studies on substandard and falsified maternal, newborn, and child health medicines and antimalarial medicines, including database-specific search terms and additional sources searched.
(DOCX)

S2 Appendix Details of the MEDQUARG quality assessment criteria. Description of the MEDQUARG-based quality assessment criteria used to appraise the methodological quality of included studies.
(DOCX)

S3 Appendix Study-level MEDQUARG methodological quality assessments. Completed 12-item MEDQUARG assessment for each study included in the meta-analysis, including the judgment or score for each quality criterion and the overall MEDQUARG score.
(DOCX)

S4 Appendix List of included studies. A numbered table of all the studies included in the meta-analysis.
(DOCX)

S5 Appendix Prevalence estimates by country and by study. Country-level pooled prevalence estimates and study-level prevalence estimates of substandard and falsified maternal, newborn, and child health medicines and antimalarial medicines across included African countries.
(DOCX)

S6 Appendix List of countries categorized by subregion. Classification of included African countries into Central, Eastern, Northern, Southern, and Western African subregions.
(DOCX)

S7 Appendix Sensitivity analysis restricted to studies with MEDQUARG score ≥6. Sensitivity analyses assessing pooled prevalence estimates after restricting the analysis to studies with MEDQUARG quality scores of 6 or higher.
(DOCX)

S8 Appendix Separate pooled prevalence estimates for substandard and falsified MNCH and antimalarial medicines. Separate pooled prevalence estimates for substandard and falsified medicines by therapeutic category, subregion, and country.
(DOCX)

S9 Appendix Meta-analysis bias and quality analysis. Additional analyses evaluating heterogeneity, publication bias, study-level influence, and moderators of substandard and falsified medicine prevalence.
(DOCX)

S10 Appendix Studies excluded from the meta-analysis. A numbered table with details of studies excluded from the quantitative synthesis because of small sample sizes, including study characteristics and bibliography.
(DOCX)

S11 Appendix Study-level data extraction dataset. Complete data extraction table for all studies included in the systematic review and meta-analysis, including study eligibility confirmation, data extractor names, dates of data extraction, study characteristics, sample counts, medicine-quality outcomes, sampling and purchasing methods, analytical tests, and the variables used in the quantitative analyses. All information was obtained directly from the included primary research reports and associated supplementary materials.
(XLSX)
