# Supplementary material for: Nearly one in five essential medicines fail quality standards in Africa: A systematic review and meta-analysis of MNCH medicines and antimalarials
Source: PLOS Glob Public Health. 2026 Sep 2;6(9):e0006326. doi: 10.1371/journal.pgph.0006326 (PMC13537570; doi:10.1371/journal.pgph.0006326)
Supplement: Supplementary file 1 [file pgph.0006326.s001.docx]

**S1 Text. Search Strategy**

**First Search**

Search for studies examining the prevalence of substandard and falsified MNCH medicines globally

Conducted: July 2^nd^, 2025

**PubMed**

| Search | Query |
| --- | --- |
| #1 | Substandard Drugs[mesh] OR Counterfeit Drugs[mesh] OR Counterfeit Medicine*[tiab] OR Counterfeit Drug*[tiab] OR Falsified Drug*[tiab] OR Fake Drug*[tiab] OR Substandard Medicine*[tiab] OR Substandard Drug*[tiab] OR Falsified Medicine*[tiab] OR Fraudulent Drug*[tiab] OR Poor-Quality Medicine*[tiab] OR Substandard Pharmaceutical*[tiab] OR counterfeit[tiab] OR substandard[tiab] OR falsified[tiab] |
| #2 | "Maternal Health"[Mesh] OR "Maternal Nutritional Physiological Phenomena"[Mesh] OR "Child Nutritional Physiological Phenomena"[Mesh:noexp] OR "Child Health"[Mesh] OR "Infant Nutritional Physiological Phenomena"[Mesh] OR "Infant Health"[Mesh] OR "Infant, Newborn"[Mesh] OR "pregnanc*"[tiab] OR "Oxytocin"[Mesh] OR "Carbetocin"[Supplementary Concept] OR "Misoprostol"[Mesh] OR "Dexamethasone"[Mesh] OR "Tranexamic acid"[Mesh] OR "Hematinics"[Mesh] OR "Folic acid"[Mesh] OR "Azithromycin"[Mesh] OR "Amoxicillin"[Mesh] OR "Gentamicins"[Mesh] OR "Ampicillin"[Mesh] OR "Artemether"[Mesh] OR "Artemisinins"[Mesh] OR "Artesunate"[Mesh] OR "BCG Vaccine"[Mesh] OR "Penicillin G"[Mesh] OR "Betamethasone"[Mesh] OR "Calcium Gluconate"[Mesh] OR "Cefixime"[Mesh] OR "Ceftriaxone"[Mesh] OR "Chlorhexidine"[Mesh] OR "Chloroquine"[Mesh] OR "Contraceptive Agents"[Mesh] OR "efavirenz, lamivudine, tenofovir disoproxil fumarate drug combination"[Supplementary Concept] OR "Hydralazine"[Mesh] OR "Artemether, Lumefantrine Drug Combination"[Mesh] OR "Magnesium Sulfate"[Mesh] OR "Measles Vaccine"[Mesh] OR "Methyldopa"[Mesh] OR "Metronidazole"[Mesh] OR "Mifepristone"[Mesh] OR "Morphine"[Mesh] OR "Nifedipine"[Mesh] OR "ORALIT"[Supplementary Concept] OR "World Health Organization oral rehydration solution" [Supplementary Concept] OR "Acetaminophen"[Mesh] OR "Pneumococcal Vaccines"[Mesh] OR "DTP-IPV-Hib vaccine"[Supplementary Concept] OR "Penicillin G Procaine"[Mesh] OR "Rotavirus Vaccines"[Mesh] OR "Sodium Lactate"[Mesh] OR "Ringer's Lactate"[Mesh] OR "Hartman's solution" [Supplementary Concept] OR "Tenofovir"[Mesh] OR "Tetanus Toxoid"[Mesh] OR "Vitamin A"[Mesh] OR "Zinc Sulfate"[Mesh] OR "Amoxicillin"[tiab] OR "Ampicillin"[tiab] OR "Artemether"[tiab] OR "Artemisinin*"[tiab] OR "Artesunate"[tiab] OR "Azithromycin"[tiab] OR "BCG Vaccine*"[tiab] OR "Penicillin G"[tiab] OR "Benzylpenicillin"[tiab] OR "Betamethasone"[tiab] OR "Calcium Gluconate"[tiab] OR "carbetocin"[tiab] OR "Cefixime"[tiab] OR "Ceftriaxone"[tiab] OR "Chlorhexidine"[tiab] OR "Chloroquine"[tiab] OR "Contraceptive Agent*"[tiab] OR "Dexamethasone"[tiab] OR "efavirenz, lamivudine, tenofovir disoproxil fumarate drug combination"[Supplementary Concept] OR "Folic Acid"[tiab] OR "Gentamicin*"[tiab] OR "Hematinic*"[tiab] OR "Hydralazine"[tiab] OR "Artemether, Lumefantrine Drug Combination"[tiab] OR "Magnesium Sulfate"[tiab] OR "Measles Vaccine*"[tiab] OR "Methyldopa"[tiab] OR "Metronidazole"[tiab] OR "Mifepristone"[tiab] OR "Misoprostol"[tiab] OR "Morphine"[tiab] OR "Nifedipine"[tiab] OR "ORS"[tiab] OR "Oral rehydration salt*"[tiab] OR "Oxytocin"[tiab] OR "Acetaminophen"[tiab] OR "Pneumococcal Vaccine*"[tiab] OR "DTP-IPV-Hib vaccine*"[tiab] OR "Penicillin G Procaine"[tiab] OR "Rotavirus Vaccine*"[tiab] OR "Sodium Lactate"[tiab] OR "Tenofovir"[tiab] OR "Tetanus Toxoid"[tiab] OR "Tetanus Toxoid"[tiab] OR "Tranexamic Acid"[tiab] OR "Vitamin A"[tiab] OR "Zinc Sulfate"[tiab] |
| #3 | #1 AND #2 |
| Total results = 327 | |

**Embase**

| Search | Query |
| --- | --- |
| #1 | ‘counterfeit drug’/exp OR (‘Substandard Drug’ OR ‘Counterfeit Drug’ OR ‘Counterfeit Medicine’ OR ‘Falsified Drug’ OR ‘Fake Drug’ OR ‘Substandard Medicine’ OR ‘Falsified Medicine*' OR ‘Fraudulent Drug*' OR ‘Poor-Quality Medicine*' OR ‘Substandard Pharmaceutical*' OR ‘counterfeit’ OR ‘substandard’ OR ‘falsified’):ab,ti,kw |
| #2 | 'maternal welfare'/exp OR 'maternal care'/exp OR 'maternal nutrition'/exp OR 'maternal and child health'/exp OR 'maternal nutrition'/exp OR 'child health'/exp OR 'child health care'/exp OR 'child nutrition'/exp OR 'pregnancy'/exp OR 'oxytocin'/exp OR 'oxytocin derivative'/exp OR 'carbetocin'/exp OR 'misoprostol'/exp OR 'mifepristone plus misoprostol'/exp OR 'dexamethasone'/exp OR 'dexamethasone derivative'/exp OR 'tranexamic acid'/exp OR 'antianemic agent'/exp OR 'folic acid'/exp OR 'azithromycin'/exp OR 'amoxicillin'/exp OR 'gentamicin'/exp OR 'ampicillin'/exp OR 'artemether'/exp OR 'artemether plus benflumetol'/exp OR 'artesunate'/exp OR 'BCG vaccine'/exp OR 'penicillin G'/exp OR 'betamethasone'/exp OR 'gluconate calcium'/exp OR 'cefixime'/exp OR 'ceftriaxone'/exp OR 'chlorhexidine'/exp OR 'chloroquine'/exp OR 'contraceptive agent'/exp OR 'efavirenz'/exp OR 'tenofovir'/exp OR 'lamivudine'/exp OR 'hydralazine'/exp OR 'magnesium sulfate'/exp OR 'measles vaccine'/exp OR 'methyldopa'/exp OR 'metronidazole'/exp OR 'mifepristone plus misoprostol'/exp OR 'mifepristone'/exp OR 'misoprostol'/exp OR 'morphine'/exp OR 'nifedipine'/exp OR 'oral rehydration solution'/exp OR 'paracetamol'/exp OR 'Pneumococcus vaccine'/exp OR 'tetanus toxoid'/exp OR 'procaine penicillin'/exp OR 'Rotavirus vaccine'/exp OR 'lactate sodium'/exp OR 'Ringer lactate solution'/exp OR 'Hartmann solution'/exp OR 'retinol'/exp OR 'zinc sulfate'/exp OR ('maternal health' OR 'maternal nutrition' OR 'child health' OR 'child nutrition' OR 'newborn*' OR 'infant*' OR 'pregnan*' OR 'oxytocin' OR 'carbetocin' OR 'misoprostol' OR 'mifepristone' OR 'dexamethasone' OR 'tranexamic acid' OR 'antianemic agent*' OR 'hematinic*' OR 'folic acid' OR 'azithromycin' OR 'amoxicillin' OR 'gentamicin*' OR 'ampicillin' OR 'artemether' OR 'artemether lumefantrine' OR 'artesunate' OR 'BCG vaccine*' OR 'penicillin G' OR 'betamethasone' OR 'gluconate calcium' OR 'cefixime' OR 'ceftriaxone' OR 'chlorhexidine' OR 'chloroquine' OR 'contraceptive*' OR 'efavirenz' OR 'tenofovir' OR 'lamivudine' OR 'hydralazine' OR 'magnesium sulfate' OR 'measles vaccine*' OR 'methyldopa' OR 'metronidazole' OR 'mifepristone' OR 'misoprostol' OR 'morphine' OR 'nifedipine' OR 'ORS' OR 'oral rehydration salt*' OR 'paracetamol' OR 'Pneumococcus vaccine*' OR 'tetanus toxoid vaccine*' OR 'procaine penicillin' OR 'penicillin G procaine' OR 'Rotavirus vaccine*' OR 'sodium lactate' OR 'Ringer lactate' OR 'Hartmann solution' OR 'retinol' OR 'zinc sulfate'):ab,ti,kw |
| #3 | #1 AND #2 |
| Total results = 1065 | |

**Web of Science**

| Search | Query |
| --- | --- |
| #1 | “Substandard Drug” OR “Counterfeit Drug” OR “Counterfeit Medicine*” OR “Falsified Drug*” OR “Fake Drug*” OR “Substandard Medicine*” OR “Substandard Drug*” OR “Falsified Medicine*” OR “Fraudulent Drug*” OR “Poor-Quality Medicine*” OR “Substandard Pharmaceutical*” OR counterfeit OR substandard OR falsified |
| #2 | “maternal health” OR “maternal nutrition” OR “child nutrition” OR “child health” OR “infant nutrition” OR “infant health” OR “infant, newborn” OR pregnanc* OR oxytocin OR carbetocin OR misoprostol OR dexamethasone OR “tranexamic acid” OR hematinics OR “folic acid” OR azithromycin OR amoxicillin OR gentamicins OR ampicillin OR artemether OR artemisinins OR artesunate OR “BCG vaccine” OR “penicillin G” OR betamethasone OR “calcium gluconate” OR cefixime OR ceftriaxone OR chlorhexidine OR chloroquine OR “contraceptive agents” OR “efavirenz, lamivudine, tenofovir disoproxil fumarate drug combination” OR hydralazine OR “artemether, lumefantrine drug combination” OR “magnesium sulfate” OR “measles vaccine” OR methyldopa OR metronidazole OR mifepristone OR morphine OR nifedipine OR ORALIT OR “World Health Organization oral rehydration solution” OR acetaminophen OR “pneumococcal vaccines” OR “DTP-IPV-Hib vaccine” OR “penicillin G procaine” OR “rotavirus vaccines” OR “sodium lactate” OR “ringer’s lactate” OR “hartman’s solution” OR tenofovir OR “tetanus toxoid” OR “vitamin A” OR “zinc sulfate” OR “artemisinin*” OR “BCG vaccine*” OR “benzylpenicillin” OR “contraceptive agent*” OR “gentamicin*” OR “hematinic*” OR “measles vaccine*” OR “ORS” OR “oral rehydration salt*” OR “pneumococcal vaccine*” OR “rotavirus vaccine*” |
| #3 | #1 AND #2 |
| Total results = 734 | |

**Second Search**

Search for studies examining the prevalence of substandard and falsified antimalarial medicines globally

Conducted: July 2^nd^, 2025

**PubMed**

| Search | Query |
| --- | --- |
| #1 | Substandard Drugs[mesh] OR Counterfeit Drugs[mesh] OR Counterfeit Medicine*[tiab] OR Counterfeit Drug*[tiab] OR Falsified Drug*[tiab] OR Fake Drug*[tiab] OR Substandard Medicine*[tiab] OR Substandard Drug*[tiab] OR Falsified Medicine*[tiab] OR Fraudulent Drug*[tiab] OR Poor-Quality Medicine*[tiab] OR Substandard Pharmaceutical*[tiab] OR counterfeit[tiab] OR substandard[tiab] OR falsified[tiab] |
| #2 | Antimalarials[Mesh] OR Anti-malarial*[tiab] OR Antimalarial*[tiab] OR Malaria[Mesh] OR Malaria[tiab] |
| #3 | #1 AND #2 |
| Total results = 221 | |

**Embase**

| Search | Query |
| --- | --- |
| #1 | ‘counterfeit drug’/exp OR (‘Substandard Drug’ OR ‘Counterfeit Drug’ OR ‘Counterfeit Medicine’ OR ‘Falsified Drug’ OR ‘Fake Drug’ OR ‘Substandard Medicine’ OR ‘Falsified Medicine*' OR ‘Fraudulent Drug*' OR ‘Poor-Quality Medicine*' OR ‘Substandard Pharmaceutical*' OR ‘counterfeit’ OR ‘substandard’ OR ‘falsified’):ab,ti,kw |
| #2 | 'antimalarial agent'/exp OR 'malaria'/exp OR ('antimalarial*' OR 'malaria'):ab,ti,kw |
| #3 | #1 AND #2 |
| Total results = 420 | |

**Web of Science**

| Search | Query |
| --- | --- |
| #1 | “Substandard Drug” OR “Counterfeit Drug” OR “Counterfeit Medicine” OR “Counterfeit Drug” OR “Falsified Drug*" OR “Fake Drug*" OR “Substandard Medicine*" OR “Substandard Drug*" OR “Falsified Medicine*" OR “Fraudulent Drug*" OR “Poor-Quality Medicine*" OR “Substandard Pharmaceutical*" OR counterfeit* OR substandard OR falsified |
| #2 | Malaria OR Antimalarial* |
| #3 | #1 AND #2 |
| Total results = 354 | |
