# Supplementary material for: Nearly one in five essential medicines fail quality standards in Africa: A systematic review and meta-analysis of MNCH medicines and antimalarials
Source: PLOS Glob Public Health. 2026 Sep 2;6(9):e0006326. doi: 10.1371/journal.pgph.0006326 (PMC13537570; doi:10.1371/journal.pgph.0006326)
Supplement: Supplementary file 2 [file pgph.0006326.s002.docx]

# **S2 Appendix. Details of the MEDQUARG Quality Assessment Criteria**

| **Item** | **Name** | **Definition** |
| --- | --- | --- |
| **1** | Survey details | The timing and location of survey (i.e. when samples were collected and analysed) are reported. |
| **2** | Definition of substandard medicines | The definitions of counterfeit, substandard and degraded medicines used by the authors are clearly provided. |
| **3** | Types of outlets | Description of the type of outlet where samples were collected is clear. This includes the agency responsible for the outlet (public, private, non-governmental), and ideally also includes information on the size of the outlet, because of medicine turnover. |
| **4** | Sampling design and sample size calculation | Clear description of the sampling design (e.g. convenience sampling, random sampling, lot quality assurance sampling—LQAS, sentinel site monitoring) is provided, as well as sample size calculation. |
| **5** | Type and dosages of units collected per outlet | Self-explanatory. |
| **6** | Random sampling method | Method of randomization used to select participating outlets. |
| **7** | Details of samplers | Information about the persons who carried out the sampling and in what guise. What did the collector say when buying the medicines? Was a mystery shopper used? |
| **8** | Packaging assessment | Description of packaging examination performed and reference standards used. |
| **9** | Statistical method | Description of the data analysis techniques used. |
| **10** | Chemical analysis | Clear description of chemical analysis and dissolution testing performed, including standard operational procedures (SOPs) and location(s) of laboratory. |
| **11** | Method validation | Details of laboratory method validation results, including but not limited to: Certificate of Analysis (COA) for reference standard, within and between run repeatability, detection and quantitation limits, accuracy observed for reference samples, linear range for all analytes, sample preparation recovery studies, selectivity. Possibly, validation against a reference method or interlaboratory study. |
| **12** | Blinding of chemical assessors | Information on whether chemical analyses were performed by investigators blinded as to the origins of the sample. |

Source: Adapted from Almuzaini T, Choonara I, Sammons H: Substandard and counterfeit medicines: a systematic review of the literature. *BMJ open* 2013, 3(8):e002923.
