# Supplementary material for: Nearly one in five essential medicines fail quality standards in Africa: A systematic review and meta-analysis of MNCH medicines and antimalarials
Source: PLOS Glob Public Health. 2026 Sep 2;6(9):e0006326. doi: 10.1371/journal.pgph.0006326 (PMC13537570; doi:10.1371/journal.pgph.0006326)
Supplement: Supplementary file 4 [file pgph.0006326.s004.docx]

# **S4 Appendix. Table 1. Studies Included on the Meta-analysis**

| **No.** | **Study** | **MEDQUARG** |
| --- | --- | --- |
| 1 | Abuga KO, Amugune BK, Ndwigah SN, Kamau FN, Thoithi GN, Ogeto JO, et al. Quality Performance of Drugs Analyzed in the Drug Analysis and Research Unit (DARU) during the Period 2006-2010. The East and Central African Journal of Pharmaceutical Sciences. 2013;16(2):33–43. | 5 |
| 2 | Abuye H, Abraham W, Kebede S, Tatiparthi R, Suleman S. Physicochemical Quality Assessment of Antimalarial Medicines: Chloroquine Phosphate and Quinine Sulfate Tablets from Drug Retail Outlets of South-West Ethiopia. Infect Drug Resist. 2020;13:691–701. | 9 |
| 3 | ACT Consortium Drug Quality Project Team; IMPACT2 Study Team. Quality of artemisinin-containing antimalarials in Tanzania’s private sector: results from a nationally representative outlet survey. American Journal of Tropical Medicine and Hygiene. 2015;92(Suppl 6):75–86. | 8 |
| 4 | Affum AO, Lowor S, Osae SD, Dickson A, Gyan BA, Tulasi D. A pilot study on quality of artesunate and amodiaquine tablets used in the fishing community of Tema, Ghana. Malaria J. 2013;12(1). | 9 |
| 5 | Ahmed F, Eticha T, Ashenef A. Quality assessment of common anti-malarial medicines marketed in Gambella, National Regional State, South Western-Ethiopia. Malaria J. 2024;23(1):278. | 11 |
| 6 | Alemie AA, Siraj EA, Wondie GB, Soboka DR, Yayehrad AT. Post-marketing quality evaluation of oxytocin injection and assessment of associated factors among selected health facilities in Addis Ababa, Ethiopia. Scientific Reports. 2024;14(1):24243. | 10 |
| 7 | Alotaibi N, Overton S, Curtis S, Nickerson JW, Attaran A, Gilmer S, et al. Toward Point-of-Care Drug Quality Assurance in Developing Countries: Comparison of Liquid Chromatography and Infrared Spectroscopy Quantitation of a Small-Scale Random Sample of Amoxicillin. American Journal of Tropical Medicine and Hygiene. 2018;99(2):477–81. | 8 |
| 8 | Alutuli L. Evaluation of the Quality of Artemether/Lumefantrine, Sulphadoxine/Pyrimethamine and Quinine Sulphate Tablets in Public and private Health Institutions in Lusaka District. 2012. | 6 |
| 9 | Ammerdorffer A, Rushwan S, Timms R, Wright P, Beeson L, Devall AJ, et al. Quality of oxytocin and tranexamic acid for the prevention and treatment of postpartum hemorrhage in Kenya, Nigeria, South Africa, and Tanzania. International Journal of Gynecology & Obstetrics. 2022;158:46–55. | 5 |
| 10 | Anjulo H, Birhane W, Hymete A, Ashenef A. Quality Assessment of Selected Essential Antimicrobial Drugs from Drug Retail Outlets of Selected Cities in Eastern Ethiopia. Am J Trop Med Hyg. 2024;110(3):596–608. | 7 |
| 11 | Anyakora C, Oni Y, Ezedinachi U, Adekoya A, Ali I, Nwachukwu C, et al. Quality medicines in maternal health: results of oxytocin, misoprostol, magnesium sulfate and calcium gluconate quality audits. BMC pregnancy and childbirth. 2018;18:1–11. | 6 |
| 12 | Ayogu EE, Ngolryeko EA, Ezeonwumelu JOC, Wawata IG, Sadiq BO. Spectrophotometric analysis of artesunate injections available in community pharmacies in Northern and Western Uganda. Future Sci OA. 2025;11(1):2511444. | 5 |
| 13 | Baba-Moussa F, Bonou J, Ahouandjinou H, Dougnon TV, Kpavode L, Raimi N, et al. Quality Control of Selected Antimalarials Sold in the Illicit Market: An Investigation Conducted in Porto-Novo City (Republic of Benin). Advances in Bioscience and Biotechnology. 2015;06(10):637–44. | 6 |
| 14 | Baratta F, Germano A, Brusa P. Diffusion of counterfeit drugs in developing countries and stability of galenics stored for months under different conditions of temperature and relative humidity. Croat Med J. 2012;53(2):173–84. | 4 |
| 15 | Bate R, Hess K. Anti-malarial drug quality in Lagos and Accra - a comparison of various quality assessments. Malaria J. 2010;9:157. | 6 |
| 16 | Bate R, Jin GZ, Mathur A. Does price reveal poor-quality drugs? Evidence from 17 countries. J Health Econ. 2011;30(6):1150–63. | 8 |
| 17 | Bate R, Hess K. The role of pre-shipment batch testing in ensuring good medicine quality. Malariaworld J. 2012;3:12. | 5 |
| 18 | Batson JS, Bempong DK, Lukulay PH, Ranieri N, Satzger RD, Verbois L. Assessment of the effectiveness of the CD3+ tool to detect counterfeit and substandard anti-malarials. Malaria J. 2016;15(1):119. | 6 |
| 19 | Bekoe SO, Ahiabu M-A, Orman E, Tersbøl BP, Adosraku RK, Hansen M, Frimodt-Møller N, Styrishave B. Exposure of consumers to substandard antibiotics from selected authorised and unauthorised medicine sales outlets in Ghana. Tropical Medicine & International Health. 2020;25(8):962–975. | 8 |
| 20 | Bekoe SO, Orman E, Adjabui SA, Brobbey AA, Oppong-Kyekyeku J, Opuni KFM, Kuntworbe N, Asare-Nkansah S. Development and validation of an ion-pair HPLC-UV method for the quantitation of quinoline and indoloquinoline alkaloids in herbal and pharmaceutical antimalarial formulations. Journal of Chemistry. 2022;2022:4625954. | 5 |
| 21 | Belew S, Suleman S, Mohammed T, Mekonnen Y, Duguma M, Teshome H, et al. Quality of fixed dose artemether/lumefantrine products in Jimma Zone, Ethiopia. Malaria J. 2019;18. | 10 |
| 22 | Bernier MC, Li F, Musselman B, Newton PN, Fernández FM. Fingerprinting of falsified artemisinin combination therapies via direct analysis in real time coupled to a compact single quadrupole mass spectrometer. Analytical Methods. 2016;8(36):6616–24. | 4 |
| 23 | Bizimana T, Hagen N, Gnegel G, Kayumba PC, Heide L. Quality of oxytocin and misoprostol in health facilities of Rwanda. PLoS One. 2021;16(1):e0245054. | 11 |
| 24 | Bizimana T, Kagisha V, Nyandwi JB, Nyirimigabo AK, Muganga R, Mukanyangezi MF, et al. Investigation of the Quality of the 12 Most-Used Antibiotics Available in Retail Private Pharmacies in Rwanda. ANTIBIOTICS-BASEL. 2022;11(3). | 10 |
| 25 | Bliese SL, Maina M, Were P, Lieberman M. Detection of degraded, adulterated, and falsified ceftriaxone using paper analytical devices. Analytical Methods. 2019;11(37):4727–32. | 5 |
| 26 | Boadu RF, Agyare C, Adarkwa-Yiadom M, Adu F, Boamah VE, Boakye YD. In vitro activity and evaluation of quality of some selected penicillins on the Ghanaian market using developed HPLC methods. Med chem. 2015;5:001–14. | 7 |
| 27 | Bower J, Chinery L, Fleurent A, Gülmezoglu AM, Im-Amornphong W, Kilfedder C, Procter P, Tomazzini A. Quality testing of mifepristone and misoprostol in 11 countries. Int J Gynecol Obstet. 2023;165(2):405–415. | 7 |
| 28 | Chikowe I, Osei-Safo D, Harrison J, Konadu DY, Addae-Mensah I. Post-marketing surveillance of anti-malarial medicines used in Malawi. Malaria J. 2015;14. | 11 |
| 29 | Chikowe I, Bliese SL, Lucas S, Lieberman M. Amoxicillin Quality and Selling Practices in Urban Pharmacies and Drug Stores of Blantyre, Malawi. The American Journal of Tropical Medicine and Hygiene. 2018;99(1):233–8. | 7 |
| 30 | Chiumia FK, Nyirongo HM, Kampira E, Muula A, Khuluza F. Burden of and factors associated with poor quality antibiotic, antimalarial, antihypertensive and antidiabetic medicines in Malawi. PLoS ONE. 2022;17. | 9 |
| 31 | Demissie D, Geremew T, Chernet AZ, Ali MM. Potency of commonly retailed antibiotics in pharmacies found in Adama, Oromia regional state, Ethiopia. PLOS ONE. 2021;16(7). | 6 |
| 32 | Denekew T, Eticha T, Teshome Y, Endeshaw S, Ashenef A. Post-marketing quality surveillance of selected antibacterial agents marketed in porous borders; the case of Ethiopia-Sudan-Eritrea border. PLoS ONE. 2024;19. | 10 |
| 33 | Djobet MN, Singhe D, Lohoue J, Kuaban C, Ngogang J, Tambo E. Antiretroviral therapy supply chain quality control and assurance in improving people living with HIV therapeutic outcomes in Cameroon. AIDS research and therapy. 2017;14(1):19. | 7 |
| 34 | Djoko E., Ngamo G.,Tekam J.M. Quality control of imidazole anti parasitical drugs distributed in Bafoussam: albendazole, mebendazole and metronidazole. International Journal of Recent Innovations in Academic Research. 2018;2(8):79–90. | 5 |
| 35 | Dohou AM, Yémoa AL, Aurel Guidan DB, Solange Ahouandjinou SH, Amoussa A, Dossou FM, et al. Assessment of the Quality of Injectable Antibiotics in Benin. American Journal of Tropical Medicine and Hygiene. 2022;107(1):24–31. | 8 |
| 36 | Ehianeta T, Williams B, Surakat J, Mohammed N, Anyakora C. Quality survey of some brands of artesunate-amodiaquine in Lagos drug market. AFRICAN JOURNAL OF PHARMACY AND PHARMACOLOGY. 2012;6(9):636–42. | 5 |
| 37 | El-Duah M, Ofori-Kwakye K. Substandard artemisinin-based antimalarial medicines in licensed retail pharmaceutical outlets in Ghana. JOURNAL OF VECTOR BORNE DISEASES. 2012;49(3):131–9. | 7 |
| 38 | Fadeyi I, Lalani M, Mailk N, Van Wyk A, Kaur H. Quality of the Antibiotics-Amoxicillin and Co-Trimoxazole from Ghana, Nigeria, and the United Kingdom. AMERICAN JOURNAL OF TROPICAL MEDICINE AND HYGIENE. 2015;92(6):87–94. | 6 |
| 39 | Fatoki S, Awodele O. Anticounterfeiting strategies of local drug manufacturers in Lagos, Nigeria: Drug safety and implications for public health. J Popul ther Clin Pharmacol. 2017;24(3):e99–e120. | 4 |
| 40 | Frimpong G, Ofori-Kwakye K, Kuntworbe N, Buabeng KO, Osei YA, Boakye-Gyasi ME, et al. Quality assessment of some essential children’s medicines sold in licensed outlets in Ashanti Region, Ghana. Journal of tropical medicine. 2018;2018(1):1494957. | 6 |
| 41 | Gabel J, Gnegel G, Kessler W, Sacré PY, Heide L. Verification of the active pharmaceutical ingredient in tablets using a low-cost near-infrared spectrometer. TALANTA OPEN. 2023;8. | 7 |
| 42 | Gabel J, Lächele M, Sander K, Gnegel G, Sunny-Abarikwu N, Ohazulike RE, et al. Quality of Essential Medicines from Different Sources in Enugu and Anambra, Nigeria. The American Journal of Tropical Medicine and Hygiene. 2024;1(aop). | 9 |
| 43 | Gnegel G, Häfele-Abah C, Neci R, Heide L. Surveillance for substandard and falsified medicines by local faith-based organizations in 13 low- and middle-income countries using the GPHF Minilab. Scientific reports. 2022;12(1):13095. | 8 |
| 44 | Hagen N, Khuluza F, Heide L. Quality, availability and storage conditions of oxytocin and misoprostol in Malawi. BMC pregnancy and childbirth. 2020;20(1):184. | 11 |
| 45 | Hajjou M, Krech L, Lane-Barlow C, Roth L, Pribluda VS, Phanouvong S, et al. Monitoring the Quality of Medicines: Results from Africa, Asia, and South America. The American Journal of Tropical Medicine and Hygiene. 2015;92(Suppl 6):68–74. | 5 |
| 46 | Hall PE, Tagontong N. Quality of misoprostol products. WHO Drug Information. 2016;30(1):35–39. | 6 |
| 47 | Hassan IA, Adegbola AJ, Soyinka JO, Onyeji CO, Bolaji OO. Post-Marketing Surveillance of Quality of Artemether Injection Marketed in Southwest Nigeria. AMERICAN JOURNAL OF TROPICAL MEDICINE AND HYGIENE. 2020;103(3):1258–65. | 9 |
| 48 | Izevbekhai O, Adeagbo B, Olagunju A, Bolaji O. Quality of artemisinin-based antimalarial drugs marketed in Nigeria. TRANSACTIONS OF THE ROYAL SOCIETY OF TROPICAL MEDICINE AND HYGIENE. 2017;111(2):90–6. | 4 |
| 49 | Jere E, Munkombwe D, Mukosha M, Mudenda S, Kalungia AC, Chabalenge B. Quality of antiretroviral, antimalarial and antituberculosis medicines in Zambia: Findings of routine post-marketing surveillance. J Med Access. 2024;8. | 6 |
| 50 | Kaale E, Manyanga V, Chambuso M, Liana J, Rutta E, Embrey M, et al. The Quality of Selected Essential Medicines Sold in Accredited Drug Dispensing Outlets and Pharmacies in Tanzania. PLOS ONE. 2016;11(11):e0165785. | 9 |
| 51 | Karikari-Boateng E, Ghana F, Boateng KP. Post-market quality surveillance project: Maternal healthcare products (oxytocin and ergometrine) on the Ghanaian market. Accra, Ghana: Ghana Food and Drugs Authority. 2013. | 5 |
| 52 | Kaur H, Allan EL, Mamadu I, et al. Quality of artemisinin-based combination formulations for malaria treatment: prevalence and risk factors for poor quality medicines in public facilities and private sector drug outlets in Enugu, Nigeria. PLoS One. 2015;10(5):e0125577. Published 2015 May 27. | 9 |
| 53 | Kaur H, Clarke S, Lalani M, Phanouvong S, Guérin P, McLoughlin A, et al. Fake anti-malarials: start with the facts. Malaria J. 2016;15:86. | 8 |
| 54 | Kaur H, Allan EL, Mamadu I, Hall Z, Green MD, Swamidos I, et al. Prevalence of substandard and falsified artemisinin-based combination antimalarial medicines on Bioko Island, Equatorial Guinea. BMJ global health. 2017;2(4):e000409. | 9 |
| 55 | Ministry of Health K. Monitoring the Quality of Antimalarial Medicines Circulating in Kenya: Round Seven. 2019. | 8 |
| 56 | Khuluza F. In-vitro evaluation of the quality of Paracetamol and Co-trimoxazole tablets used in Malawi based on pharmacopoeial standards. Malawi Medical Journal. 2014;26(2):38–41. | 8 |
| 57 | Khuluza F, Kigera S, Jähnke RWO, Heide L. Use of thin-layer chromatography to detect counterfeit sulfadoxine/pyrimethamine tablets with the wrong active ingredient in Malawi. Malaria J. 2016;15:215. | 6 |
| 58 | Khuluza F, Kigera S, Heide L. Low Prevalence of Substandard and Falsified Antimalarial and Antibiotic Medicines in Public and Faith-Based Health Facilities of Southern Malawi. The American journal of tropical medicine and hygiene. 2017;96(5):1124–35. | 9 |
| 59 | Klein EY, Lewis IA, Jung C, Llinás M, Levin SA. Relationship between treatment-seeking behaviour and artemisinin drug quality in Ghana. Malaria J. 2012;11. | 7 |
| 60 | Koech LC, Irungu BN, Ng'ang'a MM, Ondicho JM, Keter LK. Quality and Brands of Amoxicillin Formulations in Nairobi, Kenya. BIOMED RESEARCH INTERNATIONAL. 2020;2020. | 8 |
| 61 | Lambert P, Nguyen T-H, McEvoy C, Minhas RS, Wright P, Deadman K, et al. Quality of oxytocin ampoules available in health care facilities in the Democratic Republic of Congo: an exploratory study in five provinces. Journal of global health. 2018;8(2):020415. | 8 |
| 62 | Lambert P, Nguyen T-H, Oliver VL, McArthur AJ, Goodall C, Teklu AM, et al. Oxytocin injection quality in Ethiopia: a post-marketing surveillance study in public and private facilities across three regions. Journal of Global Health Reports. 2019;3:e2019081. | 6 |
| 63 | Lawal MG, Mukhtar MD, Magashi AM. Quality Assessment of Antibiotic Oral Drug Formulations Marketed In Katsina State, Nigeria. Asian Journal of Pharmaceutical Research and Development. 2019;7(6):6–10. | 3 |
| 64 | Lawson G, Ogwu J, Tanna S. Quantitative screening of the pharmaceutical ingredient for the rapid identification of substandard and falsified medicines using reflectance infrared spectroscopy. PLoS One. 2018;13(8):e0202059. | 5 |
| 65 | Lehmann A, Hofsäss M, Dressman J. Differences in drug quality between South Africa and Germany. Journal of Pharmacy and Pharmacology. 2018;70(10):1301–14. | 12 |
| 66 | Maffioli EM, Anyakora C. A comparative study between Near-Infrared (NIR) spectrometer and High-Performance Liquid Chromatography (HPLC) on the sensitivity and specificity. PloS one. 2025;20(3):e0319523. | 7 |
| 67 | Mahano AO, Mahano AZ, Cubaka NH, Kasali FM, Zirirane BB, Namegabe LM, et al. Pharmaceutical quality of antimalarial drugs: quinine sulfate and Artemether/Lumefantrine tablets sold on Bukavu Market. FUTURE JOURNAL OF PHARMACEUTICAL SCIENCES. 2021;7(1). | 6 |
| 68 | MOPHS, MOMS. Post Market Survey of First line Antiretroviral Medicines in Kenya. Republic of Kenya. Ministry of Public Health & Sanitation. Ministry of Medical Services. October 2012. Available from: <https://marketbookshelf.com/wp-content/uploads/2017/07/9150.pdf> | 12 |
| 69 | Mufusama J-P, Ndjoko Ioset K, Feineis D, Hoellein L, Holzgrabe U, Bringmann G. Quality of the antimalarial medicine artemether-lumefantrine in 8 cities of the Democratic Republic of the Congo. Drug Test Anal. 2018;10(10):1599–606. | 6 |
| 70 | Mwamba TP, Duez P, Jean-Baptiste Kalonji N. Preliminary survey of counterfeiting of albendazole and metronidazole marketed in Lubumbashi. Int J Pharm Pharm Sci. 2016;8(7):282–287. | 7 |
| 71 | Myers NM, Maina MW, Were PM, Karwa R, Pastakia SD, Sharp JC, et al. Lab on paper: assay of beta-lactam pharmaceuticals by redox titration. ANALYTICAL METHODS. 2019;11(37):4741–50. | 9 |
| 72 | Mziray S, Maganda BA, Mwamwitwa K, Fimbo AM, Kisenge S, Sambu G, et al. Quality of selected anti-retroviral medicines: Tanzania Mainland market as a case study. BMC PHARMACOLOGY & TOXICOLOGY. 2021;22(1). | 6 |
| 73 | Mziray S, Mwamwitwa K, Kisoma S, Augustine S, Fimbo A, Hipolite D, et al. Post marketing surveillance of anti-malarial medicines in Tanzania. Pharm Regul Aff. 2017;6(191):2. | 5 |
| 74 | National Agency for Food and Drug Administration and Control (NAFDAC). Report of Round Five (5) Survey of the Quality of Antimalarial Medicines in Nigeria. In: Pharmacovigiliance/Post Marketing Surveillance Newsletter: Circulation of falsified antimalarials and antibiotics in Sub-Saharan Africa. Vol. 12, No. 2. Abuja, Nigeria: NAFDAC; 2019. 2019-Vol-12-No-2-Circulation-of-falsified-antimalaria-and-antibiotics-in-subsaharan-Africa.pdf | 4 |
| 75 | Ndichu ET, Ohiri K, Sekoni O, Makinde O, Schulman K. Evaluating the quality of antihypertensive drugs in Lagos State, Nigeria. PLoS One. 2019;14(2):e0211567. | 9 |
| 76 | Ndwigah S, Stergachis A, Abuga K, Mugo H, Kibwage I. The quality of anti-malarial medicines in Embu County, Kenya. Malaria J. 2018;17. | 5 |
| 77 | Newton PN, Amin AA, Bird C, Passmore P, Dukes G, Tomson G, et al. The Primacy of Public Health Considerations in Defining Poor Quality Medicines. PLOS MEDICINE. 2011;8(12). | 5 |
| 78 | Newton PN, Chesson LA, Mayxay M, Dondorp A, Tabernero P, Howa JD, et al. Forensic investigation of falsified antimalarials using isotope ratio mass spectrometry: a pilot investigation. Scientific Reports. 2024;14(1):3995. | 6 |
| 79 | Nnanga EN, Soppo L, Dassaou Y, Nyangono N, Benga M, Ondoua S. Quality assessment of analgesics paracetamol and ibuprofen for sale in the Douala City (Cameroon). Health Sciences and Disease. 2020;21(1). | 5 |
| 80 | Nyarko S, Ofori-Kwakye K, Johnson R, Kuntworbe N, Yar DD. Investigating the Presence of Falsified and Poor‐Quality Fixed‐Dose Combination Artemether‐Lumefantrine Pharmaceutical Dosage Forms in Kumasi, Ghana. Advances in Pharmacological and Pharmaceutical Sciences. 2024;2024(1):2650540. | 9 |
| 81 | Ocan M, Nakalembe L, Otike C, Omali D, Buzibye A, Nsobya S. Pharmacopeial quality of artemether–lumefantrine anti-malarial agents in Uganda. Malaria J. 2023;22(1):165. | 8 |
| 82 | Ochekpe N, Agbowuro A, Attah S. Correlation of price and quality of medicines: assessment of some artemisinin antimalarials in Nigeria based on GPHF minilab. Int J Drug Dev Res. 2010;2(1):211–8. | 7 |
| 83 | Odunfa O, Adegoke O, Onaga I. Pharmaceutical equivalence of some commercial samples of Artesunate and Amodiaquine tablets sold in Southwestern Nigeria. Tropical Journal of Pharmaceutical Research. 2009;8(6). | 7 |
| 84 | Oli AN, Nwankwo EJ, Umeyor CE, Umeh US, Okoyeh JN, Ofomata CM, et al. Emergency medicine: magnesium sulphate injections and their pharmaceutical quality concerns. Heliyon. 2021;7(5). | 8 |
| 85 | Omer R, Abdelatti N, Khalid A, Abakar D, Fadul A, Osman E, et al. Development and Validation of Direct UV-Spectrophotometric Methods for the Analysis of Folic Acid in Bulk and Dosage Form. Journal of Applied Spectroscopy. 2024;91(5):1141–8. | 4 |
| 86 | Onah CM, Obelu AD, Nnadi MC, Iloegbunam JC, Akpan IN, Ozioko KC, et al. Monitoring the quality of quinine injection in retail outlets in south-east Nigeria. Tropical Journal of Pharmaceutical Research. 2025;24(5). | 10 |
| 87 | Opoku S, Nyanor I. Qualitative and Quantitative Microbiological Studies of Paediatric Artemether-Lumefantrine Dry Powders and Paracetamol Syrups Obtained from Selected Drug Stores in Accra, Ghana. JOURNAL OF TROPICAL MEDICINE. 2019;2019. | 6 |
| 88 | Opuni KF-M, Nettey H, Larbi MA, Amartey SNA, Nti G, Dzidonu A, et al. Usefulness of combined screening methods for rapid detection of falsified and/or substandard medicines in the absence of a confirmatory method. Malaria J. 2019;18(1):403. | 5 |
| 89 | Opuni KFM, Sunkwa-Mills G, Antwi MA, Squire A, Afful GY, de Wit TFR, et al. Quality assessment of medicines in selected resource-limited primary healthcare facilities using low- to medium-cost field testing digital technologies. DIGITAL HEALTH. 2024;10. | 7 |
| 90 | Oresanya AO, Ayodele AO, Awotuya OI, Fagbohun BA, Olufolabo OK, Daodu OJ, et al. Chemical analysis and antibacterial susceptibility of some brands of amoxicillin suspension marketed in Sagamu, Ogun, Nigeria. Vietnam Journal of Chemistry. 2025;63(5):685–92. | 6 |
| 91 | Osei-Safo D, Agbonon A, Konadu DY, Harrison JJEK, Edoh M, Gordon A, et al. Evaluation of the Quality of Artemisinin‐Based Antimalarial Medicines Distributed in Ghana and Togo. Malaria Research and Treatment. 2014;2014(1):806416. | 8 |
| 92 | Osei-Safo D, Egbo HA, Nettey H, Konadu DY, Addae-Mensah I. Evaluation of the quality of some antibiotics distributed in Accra and Lagos. International Journal of Pharmaceutical Sciences and Research. 2016;7(5):1991–2000. | 8 |
| 93 | Patel A, Gauld R, Norris P, Rades T. Quality of generic medicines in South Africa: Perceptions versus Reality – A qualitative study. BMC Health Services Research. 2012;12(1):297. | 7 |
| 94 | Petersen A, Held N, Heide L, Difam EPNMSG. Surveillance for falsified and substandard medicines in Africa and Asia by local organizations using the low-cost GPHF Minilab. PLoS One. 2017;12(9):e0184165. | 5 |
| 95 | Prah J, Ameyaw EO, Afoakwah R, Fiawoyife P, Oppong-Danquah E, Boampong JN. Quality Assessment of Artemether‐Lumefantrine Samples and Artemether Injections Sold in the Cape Coast Metropolis. Journal of Tropical Medicine. 2016;2016(1):8602619. | 9 |
| 96 | Sakira AK, Mees C, De Braekeleer K, Delporte C, Yameogo J, Yabre M, et al. Determination of the quality of metronidazole formulations by near-infrared spectrophotometric analysis. TALANTA OPEN. 2021;3. | 5 |
| 97 | Sawadogo C, Amood Al-Kamarany M, Al-Mekhlafi H, Elkarbane M, Al-Adhroey A, Cherrah Y, et al. Quality of chloroquine tablets available in Africa. Annals of Tropical Medicine & Parasitology. 2011;105(6):447–53. | 4 |
| 98 | Schäfermann S, Wemakor E, Hauk C, Heide L. Quality of medicines in southern Togo: investigation of antibiotics and of medicines for non-communicable diseases from pharmacies and informal vendors. PloS one. 2018;13(11):e0207911. | 10 |
| 99 | Schäfermann S, Hauk C, Wemakor E, Neci R, Mutombo G, Ndze EN, et al. Substandard and falsified antibiotics and medicines against noncommunicable diseases in western Cameroon and northeastern Democratic Republic of Congo. The American Journal of Tropical Medicine and Hygiene. 2020;103(2):894. | 10 |
| 100 | Schiavetti B, Wynendaele E, De Spiegeleer B, Mbinze GJ, Kalenda N, Marini R, et al. The Quality of Medicines Used in Children and Supplied by Private Pharmaceutical Wholesalers in Kinshasa, Democratic Republic of Congo: A Prospective Survey. The American journal of tropical medicine and hygiene. 2018;98(3):894–903. | 8 |
| 101 | Soppo Lobe V, Nnanga Nga N, Nyangono Ndongo M, Nko’o MJ, Ngo Maniben P, Ngolsou F, Betoté DPH, Benga Mekoulou C, Maniepi NPJS, Dimaïssou JA, Mpondo Mpondo E, Ze Minkande J. Quality of the ceftriaxone in the formal circuit of the city of Yaoundé. Health Sciences and Disease. 2020;21(11):59–64. Available from: <https://www.hsd-fmsb.org/index.php/hsd/article/view/2377/pdf_1099> | 7 |
| 102 | Stanton C, Koski A, Cofie P, Mirzabagi E, Grady BL, Brooke S. Uterotonic drug quality: an assessment of the potency of injectable uterotonic drugs purchased by simulated clients in three districts in Ghana. BMJ open. 2012;2(3):e000431. | 8 |
| 103 | Tchounga CAW, Marini D, Nga EN, Hamuli PC, Mballa RN, Hubert P, et al. In-Field Implementation of Near-Infrared Quantitative Methods for Analysis of Medicines in Tropical Environments. APPLIED SPECTROSCOPY. 2023;77(11):1264–79. | 11 |
| 104 | Teklu L, Adugna E, Ashenef A. Quality evaluation of paracetamol tablets obtained from the common shops (kiosks) in addis ababa, ethiopia. International journal of pharmaceutical sciences and research. 2014;5(8):3502–10. | 6 |
| 105 | Tivura M, Asante I, van Wyk A, et al. Quality of Artemisinin-based Combination Therapy for malaria found in Ghanaian markets and public health implications of their use. BMC Pharmacol Toxicol. 2016;17:48. | 10 |
| 106 | Tshilombo N, Hamuli P, Mbinze J, Habyalimana V, Kalenda D, Mavungu D, Mwamba P, Hubert P, Marini RD. Investigation of the quality of antibiotics-based amoxicillin for monitoring of some different medicine markets of Democratic Republic of Congo. American Journal of Analytical Chemistry. 2018;9:366–385. | 9 |
| 107 | Tshilumba PM, Amuri SB, Kaghowa ER, Mbikay DM, Impele AB, Duez P, et al. Survey of counterfeit oral antiinfectives medecine sold in Lubumbashi. The Pan African Medical Journal. 2015;22:318. | 5 |
| 108 | Tshilumba PM, Kagoha ER, Chiribagula VB, Kalubandika GM, Ndage VN, Nzuzi TS, Wa Ilunga E, Duez P, Kalonji Ndoumba JB. Preliminary study on counterfeiting of artemether and artesunate marketed in Lubumbashi. Pharmacology & Pharmacy. 2016;7(5):185–192. | 7 |
| 109 | Tshilumba PM, Ilangala AB, Kindenge JM, Kasongo IM, Kikunda G, Rongorongo E, et al. Detection of Substandard and Falsified Antibiotics Sold in the Democratic Republic of the Congo Using Validated HPLC and UV-Visible Spectrophotometric Methods. American Journal of Tropical Medicine and Hygiene. 2023;109(2):480–8. | 10 |
| 110 | Visser BJ, Meerveld-Gerrits J, Kroon D, Mougoula J, Vingerling R, Bache E, et al. Assessing the quality of anti-malarial drugs from Gabonese pharmacies using the MiniLab®: a field study. Malaria J. 2015;14(1):273. | 9 |
| 111 | Wafula F, Dolinger A, Daniels B, Mwaura N, Bedoya G, Rogo K, et al. Examining the quality of medicines at Kenyan healthcare facilities: a validation of an alternative post-market surveillance model that uses standardized patients. Drugs-real world outcomes. 2016;4:53–63. | 6 |
| 112 | Wang T, Hoag SW, Eng ML, Polli J, Pandit NS. Quality of antiretroviral and opportunistic infection medications dispensed from developing countries and Internet pharmacies. Journal of clinical pharmacy and therapeutics. 2015;40(1):68–75. | 8 |
| 113 | Ward K, Suleiman R, Kippie Y, Dube A. A post-market quality assessment of first-line, fixed-dose combination antiretrovirals in South Africa. Journal of Applied Pharmaceutical Science. 2019;9(2):097–104. | 6 |
| 114 | World Health Organization. Quality of antimalarial medicines in sub-Saharan Africa (QAMSA). Geneva: World Health Organization. 2011. Available from: <https://www.afro.who.int/sites/default/files/2017-06/WHO_QAMSA_report.pdf> | 11 |
| 115 | World Health Organization. Survey of the quality of medicines identified by the United Nations Commission on Life-Saving Commodities for Women and Children. Geneva: World Health Organization; 2016. Available from: <https://www.who.int/publications/i/item/survey-of-the-quality-of-medicines-identified-by-the-united-nations-commission-on-life-saving-commodities-for-women-and-children> | 9 |
| 116 | World Health Organization. Survey of the quality of selected antiretroviral medicines circulating in five African countries. Geneva: World Health Organization; 2019. Available from: <https://www.who.int/publications/i/item/WHO-EMP-RHT-RSS-2019.01> | 6 |
| 117 | Wilson BK, Kaur H, Allan EL, Lozama A, Bell D. A new handheld device for the detection of falsified medicines: demonstration on falsified artemisinin-based therapies from the field. The American journal of tropical medicine and hygiene. 2017;96(5):1117. | 6 |
| 118 | Wynendaele E, Colpaert M, Debunne N, Kosgei A, De Spiegeleer B. Residual active pharmaceutical ingredient on the primary packaging of medicines: A valuable GMP-marker. Talanta. 2019;201:259–65. | 7 |
| 119 | Yabré M, Ferey L, Sakira AK, Bonmatin C, Fauré C, Somé TI, Gaudin K. Green analytical methods of antimalarial artemether-lumefantrine analysis for falsification detection using a low-cost handheld NIR spectrometer with DD-SIMCA and drug quantification by HPLC. Molecules. 2020;25(15):3397. | 7 |
| 120 | Yabré M, Sakira AK, Bandé M, Goumbri BWF, Ouattara SM, Fofana S, Somé TI. Detection of falsified antimalarial sulfadoxine–pyrimethamine and dihydroartemisinin–piperaquine drugs using a low-cost handheld near-infrared spectrometer. Journal of Analytical Methods in Chemistry. 2022;2022:5335936. | 7 |
| 121 | Yabré M, Sakira AK, Bandé M, Delma KL, Nyamba I, Yemoa LY A, Fofana S, Somé TI. Qualitative and quantitative analysis of metronidazole formulations using a low-cost near infrared transmission spectrophotometer and chemometric tools. Anal Methods. 2025;17:2312–2320. | 5 |
| 122 | Yemoa A, Habyalimana V, K. Mbinze J, Crickboom V, Muhigirwa B, Ngoya A, et al. Detection of poor quality artemisinin-based combination therapy (ACT) medicines marketed in Benin using simple and advanced analytical techniques. Current Drug Safety. 2017;12(3):178–86. | 6 |
| 123 | Yemoa AL, Assanhou AG, Agbokponto JE, Adebo M, Ganfon H, Gbaguidi FA. Post-market surveillance (PMS) of artemether-lumefantrine fixed-dose combinations marketed in Benin. Int J Pharm Sci & Res. 2022. | 6 |

Data extraction was conducted by: M.D.N
