# Supplementary material for: Nearly one in five essential medicines fail quality standards in Africa: A systematic review and meta-analysis of MNCH medicines and antimalarials
Source: PLOS Glob Public Health. 2026 Sep 2;6(9):e0006326. doi: 10.1371/journal.pgph.0006326 (PMC13537570; doi:10.1371/journal.pgph.0006326)
Supplement: Supplementary file 5 [file pgph.0006326.s005.docx]

# **S1 File. Prevalence estimates by country and by study.**

## **S1 File. Figure 1. Pooled prevalence of Substandard and Falsified MNCH and antimalarial Medicines by Country in 34 African countries from studies published between 2010-2025.**

***Note****: Country labeled “N/S” represents study Bernier et al. (2016) which collected samples in “Sub-Saharan African countries”, however the exact countries are not stated by the authors.*

## **S1 File. Figure 2. Results from Random-Effects Meta-Analysis of Substandard and Falsified Essential MNCH and Antimalarial Medicines in 34 African countries from studies published between 2010-2025.**
