# Supplementary material for: Nearly one in five essential medicines fail quality standards in Africa: A systematic review and meta-analysis of MNCH medicines and antimalarials
Source: PLOS Glob Public Health. 2026 Sep 2;6(9):e0006326. doi: 10.1371/journal.pgph.0006326 (PMC13537570; doi:10.1371/journal.pgph.0006326)
Supplement: Supplementary file 6 [file pgph.0006326.s006.docx]

# **S4 Table. List of countries categorized by subregion.**

| **Region** | **Country** |
| --- | --- |
| **Central Africa** | Cameroon |
|  | Central African Republic |
|  | Chad |
|  | DR Congo |
|  | Equatorial Guinea |
|  | Gabon |
| **Eastern Africa** | Burundi |
|  | Comoros |
|  | Ethiopia |
|  | Kenya |
|  | Madagascar |
|  | Malawi |
|  | Rwanda |
|  | Tanzania |
|  | Uganda |
| **Northern Africa** | Egypt |
|  | Morocco |
|  | Sudan |
| **Southern Africa** | Angola |
|  | Mozambique |
|  | South Africa |
|  | Zambia |
|  | Zimbabwe |
| **Western Africa** | Benin |
|  | Burkina Faso |
|  | Ghana |
|  | Ivory Coast |
|  | Liberia |
|  | Mali |
|  | Niger |
|  | Nigeria |
|  | Senegal |
|  | Sierra Leone |
|  | Togo |
