# Supplementary material for: Nearly one in five essential medicines fail quality standards in Africa: A systematic review and meta-analysis of MNCH medicines and antimalarials
Source: PLOS Glob Public Health. 2026 Sep 2;6(9):e0006326. doi: 10.1371/journal.pgph.0006326 (PMC13537570; doi:10.1371/journal.pgph.0006326)
Supplement: Supplementary file 7 [file pgph.0006326.s007.docx]

# **S2 File. Sensitivity Analysis Restricted to Studies with MEDQUARG ≥6**

## **S2 File. Figure 1. Random-Effects Meta-Analysis of Substandard and Falsified Essential MNCNH and Antimalarial Medicines in 25 African Countries (2010–2025), Restricted to Studies with MEDQUARG ≥6**

## **S2 File. Figure 2. Pooled prevalence of Substandard and Falsified MNCH and antimalarial Medicines by Country in 25 African countries from studies published between 2010-2025, Restricted to Studies with MEDQUARG ≥6**

## **S2 File. Figure 3. Pooled prevalence of Substandard and Falsified MNCH and antimalarial Medicines by Subregion in 25 African countries from studies published between 2010-2025, Restricted to Studies with MEDQUARG ≥6**

## **S2 File. Figure 4. Pooled prevalence of Substandard and Falsified MNCH and antimalarial Medicines by Therapeutic Category in 25 African countries from studies published between 2010-2025, Restricted to Studies with MEDQUARG ≥6**
