# Supplementary material for: Nearly one in five essential medicines fail quality standards in Africa: A systematic review and meta-analysis of MNCH medicines and antimalarials
Source: PLOS Glob Public Health. 2026 Sep 2;6(9):e0006326. doi: 10.1371/journal.pgph.0006326 (PMC13537570; doi:10.1371/journal.pgph.0006326)
Supplement: Supplementary file 8 [file pgph.0006326.s008.docx]

# **S3 File. Separate Pooled Prevalence Estimates for Substandard and Falsified MNCH**

## **S3 File. Table 1. Separate Pooled Prevalence Estimates for Substandard and Falsified MNCH and Antimalarial Medicines by Therapeutic Category, Subregion in 34 African countries (2010–2025)**

|  | **# Falsified Samples** | **Prevalence (95% CI)** | **# Substandard Samples** | **Prevalence (95% CI)** |
| --- | --- | --- | --- | --- |
| **Therapeutic Category** | | | | |
| Analgesic | 24 | 0.2% (0.0%–2.4%) | 42 | 1.6% (0.0%–5.3%) |
| Antibiotic | 59 | 0.3% (0.0%–1.4%) | 540 | 11.7% (5.6%–19.3%) |
| Anticonvulsant | 4 | 0.2% (0.0%–2.9%) | 0 | 0.0% (0.0%–0.0%) |
| Antidiarrheal | 0 | 0.0% (0.0%–8.8%) | 0 | 0.0% (0.0%–8.8%) |
| Antifibrinolytic | 0 | 0.0% (0.0%–11.9%) | 0 | 0.0% (0.0%–11.9%) |
| Antihypertensive | 22 | 8.6% (0.0%–48.9%) | 30 | 4.0% (0.0%–21.4%) |
| Antimalarial | 696 | 2.1% (0.7%–4.0%) | 2300 | 11.9% (6.8%–18.0%) |
| Antiprogestogen | 0 | 0.0% (0.0%–69.7%) | 1 | 50.0% (0.0%–100.0%) |
| Antiretroviral | 16 | 0.0% (0.0%–0.0%) | 1 | 0.0% (0.0%–0.0%) |
| Contraceptive | 0 | 0.0% (0.0%–13.9%) | 0 | 0.0% (0.0%–13.9%) |
| Corticosteroid | 0 | 0.0% (0.0%–5.5%) | 23 | 65.0% (6.2%–100.0%) |
| Electrolyte | 10 | 0.0% (0.0%–1.7%) | 0 | 0.0% (0.0%–0.0%) |
| Hematinic | 5 | 4.8% (0.0%–37.0%) | 4 | 20.0% (0.0%–88.3%) |
| Uterotonic | 160 | 0.1% (0.0%–4.3%) | 398 | 37.1% (16.4%–60.1%) |
| **Subregion** | | | | |
| Central Africa | 221 | 3.9% (0.6%–8.9%) | 428 | 10.0% (3.7%–18.2%) |
| Eastern Africa | 152 | 0.0% (0.0%–0.1%) | 1282 | 0.0% (0.0%–0.9%) |
| N/S (Sub-Sahara) | 6 | 3.1% (1.1%–6.1%) | 0 | 6.8% (3.0%–11.8%) |
| Northern Africa | 3 | 0.0% (0.0%–6.1%) | 13 | 4.8% (0.0%–25.0%) |
| Southern Africa | 53 | 0.0% (0.0%–0.8%) | 59 | 0.0% (0.0%–1.6%) |
| Western Africa | 561 | 1.4% (0.4%–2.7%) | 1557 | 15.5% (9.3%–22.7%) |
| **Country** | | | | |
| Liberia | 46 | 0.0% (0.0%–0.2%) | 1 | 0.0% (0.0%–0.0%) |
| Angola | 5 | 8.0% (0.0%–50.5%) | 7 | 0.9% (0.0%–5.4%) |
| Benin | 7 | 1.0% (0.0%–9.2%) | 17 | 7.2% (0.0%–21.6%) |
| Burkina Faso | 31 | 0.6% (0.0%–5.8%) | 4 | 0.0% (0.0%–4.3%) |
| Burundi | 0 | 0.0% (0.0%–1.5%) | 0 | 0.0% (0.0%–1.5%) |
| Cameroon | 24 | 1.0% (0.0%–5.1%) | 26 | 4.1% (0.0%–12.6%) |
| Central African Republic | 3 | 12.5% (1.7%–29.3%) | 0 | 0.0% (0.0%–7.0%) |
| Chad | 4 | 0.0% (0.0%–7.5%) | 0 | 0.0% (0.0%–0.0%) |
| Comoros | 0 | 0.0% (0.0%–100.0%) | 0 | 0.0% (0.0%–100.0%) |
| DR Congo | 52 | 5.3% (0.2%–14.3%) | 286 | 15.7% (7.0%–26.6%) |
| Egypt | 3 | 3.2% (0.0%–19.4%) | 11 | 9.8% (0.0%–33.9%) |
| Equatorial Guinea | 130 | 6.3% (4.5%–8.5%) | 40 | 1.9% (1.3%–2.6%) |
| Ethiopia | 48 | 0.0% (0.0%–1.2%) | 78 | 3.8% (0.1%–10.6%) |
| Gabon | 8 | 0.0% (0.0%–0.0%) | 70 | 9.4% (5.7%–13.5%) |
| Ghana | 104 | 0.2% (0.0%–1.4%) | 741 | 18.4% (7.7%–31.9%) |
| Ivory Coast | 0 | 0.0% (0.0%–100.0%) | 0 | 0.0% (0.0%–100.0%) |
| Kenya | 79 | 0.7% (0.0%–3.5%) | 141 | 3.2% (0.0%–10.7%) |
| Madagascar | 0 | 0.0% (0.0%–11.2%) | 1 | 6.7% (0.0%–26.4%) |
| Malawi | 2 | 0.0% (0.0%–0.3%) | 139 | 11.8% (0.0%–39.6%) |
| Mali | 0 | 0.0% (0.0%–50.0%) | 0 | 0.0% (0.0%–50.0%) |
| Morocco | 0 | 0.0% (0.0%–100.0%) | 0 | 0.0% (0.0%–100.0%) |
| Mozambique | 46 | 7.9% (5.8%–10.2%) | 1 | 0.2% (0.0%–0.7%) |
| N/S | 6 | 3.1% (1.1%–6.1%) | 0 | 0.0% (0.0%–0.9%) |
| Niger | 0 | 0.0% (0.0%–50.0%) | 1 | 33.3% (0.0%–94.1%) |
| Nigeria | 373 | 1.6% (0.1%–4.1%) | 741 | 12.2% (8.2%–16.8%) |
| Rwanda | 0 | 0.0% (0.0%–0.0%) | 50 | 5.8% (1.0%–12.9%) |
| Senegal | 0 | 0.0% (0.0%–100.0%) | 0 | 0.0% (0.0%–100.0%) |
| Sierra Leone | 0 | 0.0% (0.0%–0.0%) | 3 | 0.0% (0.0%–0.9%) |
| South Africa | 0 | 0.0% (0.0%–0.0%) | 15 | 0.0% (0.0%–1.2%) |
| Sudan | 0 | 0.0% (0.0%–8.4%) | 2 | 10.0% (0.2%–27.8%) |
| Tanzania | 20 | 0.0% (0.0%–0.0%) | 832 | 3.6% (0.0%–13.5%) |
| Togo | 0 | 0.0% (0.0%–0.0%) | 55 | 16.7% (0.0%–68.5%) |
| Uganda | 3 | 0.0% (0.0%–1.7%) | 41 | 11.5% (0.9%–28.9%) |
| Zambia | 2 | 0.0% (0.0%–2.1%) | 36 | 1.8% (0.0%–7.5%) |
| Zimbabwe | 0 | 0.0% (0.0%–11.2%) | 0 | 0.0% (0.0%–11.2%) |
| **Total** | **996** | **1.2% (0.5%–2.1%)** | **3339** | **12.8% (8.9%–17.2%)** |

*Note: Country labeled “N/S” represents study Bernier et al. (2016) which collected samples in “Sub-Saharan African countries”, however the exact countries are not stated by the authors.*

## **S3 File. Table 2. Separate Pooled Prevalence Estimates for Substandard and Falsified MNCH and Antimalarial Medicines by Quality Classification in 25 African Countries (2010–2025), Restricted to Studies with MEDQUARG ≥6**

|  | **# Falsified Samples** | **Prevalence (95% CI)** | **# Substandard Samples** | **Prevalence (95% CI)** |
| --- | --- | --- | --- | --- |
| **Therapeutic Category** | | | | |
| Analgesic | 20 | 0.1% (0.0%–2.5%) | 42 | 2.9% (0.0%–8.3%) |
| Antibiotic | 17 | 0.0% (0.0%–0.1%) | 513 | 14.9% (7.0%–24.8%) |
| Anticonvulsant | 4 | 0.2% (0.0%–2.9%) | 0 | 0.0% (0.0%–0.0%) |
| Antidiarrheal | 0 | 0.0% (0.0%–8.8%) | 0 | 0.0% (0.0%–8.8%) |
| Antihypertensive | 22 | 8.6% (0.0%–48.9%) | 30 | 4.0% (0.0%–21.4%) |
| Antimalarial | 449 | 2.3% (0.5%–4.7%) | 2146 | 11.6% (5.9%–18.6%) |
| Antiprogestogen | 0 | 0.0% (0.0%–69.7%) | 1 | 50.0% (0.0%–100.0%) |
| Antiretroviral | 0 | 0.0% (0.0%–0.2%) | 0 | 0.0% (0.0%–0.2%) |
| Contraceptive | 0 | 0.0% (0.0%–18.3%) | 0 | 0.0% (0.0%–18.3%) |
| Corticosteroid | 0 | 0.0% (0.0%–5.5%) | 23 | 65.0% (6.2%–100.0%) |
| Electrolyte | 10 | 2.7% (0.1%–7.3%) | 0 | 0.0% (0.0%–0.0%) |
| Hematinic | 5 | 21.9% (2.9%–48.0%) | 2 | 33.8% (0.0%–100.0%) |
| Uterotonic | 155 | 1.8% (0.0%–8.1%) | 208 | 36.4% (13.7%–62.6%) |
| **Subregion** | | | | |
| Central Africa | 199 | 4.0% (0.3%–10.2%) | 412 | 12.7% (4.5%–23.6%) |
| Eastern Africa | 17 | 0.0% (0.0%–0.0%) | 1218 | 8.0% (3.5%–13.9%) |
| Northern Africa | 3 | 10.1% (0.6%–25.6%) | 4 | 15.1% (0.0%–81.5%) |
| Southern Africa | 7 | 0.0% (0.0%–1.0%) | 45 | 0.0% (0.0%–0.3%) |
| **Country** | | | | |
| Angola | 5 | 20.9% (2.6%–46.2%) | 0 | 0.0% (0.0%–4.2%) |
| Benin | 7 | 3.6% (0.0%–14.0%) | 17 | 11.6% (1.1%–28.8%) |
| Burkina Faso | 31 | 2.0% (0.0%–10.7%) | 0 | 0.0% (0.0%–0.0%) |
| Burundi | 0 | 0.0% (0.0%–1.5%) | 0 | 0.0% (0.0%–1.5%) |
| Cameroon | 12 | 0.6% (0.0%–4.3%) | 25 | 9.1% (0.0%–35.6%) |
| Central African Republic | 3 | 12.5% (1.7%–29.3%) | 0 | 0.0% (0.0%–7.0%) |
| Chad | 4 | 8.3% (1.9%–18.1%) | 0 | 0.0% (0.0%–3.6%) |
| DR Congo | 42 | 5.4% (0.0%–16.6%) | 277 | 20.1% (7.0%–36.9%) |
| Egypt | 3 | 10.1% (0.6%–25.6%) | 4 | 15.1% (0.0%–81.5%) |
| Equatorial Guinea | 130 | 6.3% (4.5%–8.5%) | 40 | 1.9% (1.3%–2.6%) |
| Ethiopia | 2 | 0.0% (0.0%–0.0%) | 70 | 4.2% (0.3%–11.0%) |
| Gabon | 8 | 1.9% (0.8%–3.4%) | 70 | 16.2% (12.9%–19.8%) |
| Ghana | 52 | 0.8% (0.0%–2.9%) | 541 | 20.0% (7.9%–35.4%) |
| Kenya | 6 | 0.2% (0.0%–3.1%) | 127 | 5.7% (0.0%–19.3%) |
| Madagascar | 0 | 0.0% (0.0%–11.2%) | 1 | 6.7% (0.0%–26.4%) |
| Malawi | 2 | 0.0% (0.0%–0.3%) | 139 | 11.9% (0.0%–35.2%) |
| Nigeria | 366 | 2.2% (0.2%–5.5%) | 670 | 13.4% (5.0%–24.4%) |
| Rwanda | 0 | 0.0% (0.0%–0.0%) | 43 | 6.1% (0.3%–15.9%) |
| Sierra Leone | 0 | 0.0% (0.0%–1.6%) | 3 | 2.9% (0.4%–7.1%) |
| South Africa | 0 | 0.0% (0.0%–0.0%) | 15 | 0.0% (0.0%–3.1%) |
| Tanzania | 4 | 0.0% (0.0%–0.7%) | 819 | 5.6% (1.0%–13.2%) |
| Togo | 0 | 0.0% (0.0%–1.2%) | 55 | 23.1% (0.0%–73.3%) |
| Uganda | 3 | 0.3% (0.0%–6.6%) | 19 | 4.8% (0.0%–17.1%) |
| Zambia | 2 | 0.1% (0.0%–4.1%) | 30 | 0.2% (0.0%–2.1%) |
| Zimbabwe | 0 | 0.0% (0.0%–11.2%) | 0 | 0.0% (0.0%–11.2%) |
| **Total** | **682** | **1.1% (0.3%–2.2%)** | **2965** | **13.2% (8.7%–18.4%)** |
