# Supplementary material for: Nearly one in five essential medicines fail quality standards in Africa: A systematic review and meta-analysis of MNCH medicines and antimalarials
Source: PLOS Glob Public Health. 2026 Sep 2;6(9):e0006326. doi: 10.1371/journal.pgph.0006326 (PMC13537570; doi:10.1371/journal.pgph.0006326)
Supplement: Supplementary file 9 [file pgph.0006326.s009.docx]

# **S4 File. Meta-analysis Bias and Quality Analysis**

Given the wide variation across studies in sampling approaches, testing methods, medicine types, geographic coverage, and reporting quality, substantial differences in reported prevalence of SF medicines were anticipated. To account for this variability, we conducted a random-effects meta-analysis using prevalence estimates from the 123 unique studies. Heterogeneity was evaluated using Cochran’s Q, τ², τ, I², and H statistics. Potential publication bias was assessed through visual inspection of funnel plots and a regression-based test for asymmetry, while study-level influence was examined using Baujat plots and influence diagnostics. To explore which study characteristics might help explain the variation in SF prevalence across studies, we evaluated factors including sample size, publication year, therapeutic category, subregion, MEDQUARG score, use of random sampling, and use of mystery-client purchasing through mixed-effects meta-regression models.

**Heterogeneity:** The analysis revealed extremely high heterogeneity, indicating that reported SF prevalence varied far more than would be expected from sampling error alone. Cochran’s Q statistic was Q(df = 122) = 5671.83, p < 0.0001. The estimated between-study variance (τ²) was 0.0427, corresponding to a between-study standard deviation (τ) of 0.2066. The I² statistic was 97.8%, meaning that nearly all observed variability reflects real differences across studies rather than random variation. The H statistic was 6.82, further indicating substantial dispersion in study findings. Collectively, these measures confirm pronounced heterogeneity in the evidence base and support the use of random-effects modeling and meta-regression to investigate study-level characteristics associated with differences in reported SF prevalence.

**Influence**: Influence analyses identified five studies—Gabel et al., 2023 [66]; Chikowe et al., 2015 [53]; Wynendaele et al., 2019 [141]; Stanton et al., 2012 [125]; and Bekoe et al., 2020 [44]—as having the greatest impact on the pooled prevalence estimates and overall heterogeneity. These studies showed the largest Cook’s distances and appeared as prominent outliers across the Baujat plot and multi-panel influence diagnostics (including *rstudent*, *dffits*, *cook.d*, and *QE.del*). However, sequentially excluding each study did not materially alter the pooled prevalence or the heterogeneity statistics, indicating that no single study disproportionately influenced the meta-analytic findings.

**Publication bias:** To assess potential publication bias, we constructed a funnel plot of study estimates against their standard errors. Visual inspection indicated clear asymmetry, with smaller studies tending to report higher proportions of substandard or falsified medicines. A regression test for funnel plot asymmetry was conducted and yielded a statistically significant result (t = 3.78, df = 121, p = 0.0002), indicating evidence of small-study effects.

**Moderator**: Therapeutic category, subregion and Mystery client approach were the only significant factors.

## **Heterogeneity**

## **Publication Bias**

**Funnel Plot**

## **Individual Study Influence on Heterogeneity:**

**Baujat Plot**

## **Influence Plot and Output**

|  | rstudent | dffits | cook.d | cov.r | tau2.del | QE.del | hat | weight | inf |
| --- | --- | --- | --- | --- | --- | --- | --- | --- | --- |
| 1 | 0.70628 | 0.080328 | 0.006197 | 0.9736 | 0.040991 | 5150.003 | 0.009209 | 0.920942 |  |
| 2 | 0.019629 | 0.000938 | 8.82E-07 | 1.011864 | 0.042824 | 5662.423 | 0.008871 | 0.887069 |  |
| 3 | -1.76435 | -0.16246 | 0.026378 | 1.007942 | 0.042659 | 5656.064 | 0.008425 | 0.842488 |  |
| 4 | -1.03901 | -0.09259 | 0.00858 | 1.0088 | 0.042728 | 5670.332 | 0.00783 | 0.782951 |  |
| 5 | -1.27912 | -0.11742 | 0.0138 | 1.009349 | 0.042731 | 5666.935 | 0.008313 | 0.831307 |  |
| 6 | -0.75622 | -0.07214 | 0.005223 | 1.012192 | 0.042844 | 5671.304 | 0.008763 | 0.876304 |  |
| 7 | -1.21391 | -0.11725 | 0.013818 | 1.013894 | 0.042913 | 5656.204 | 0.009016 | 0.901624 |  |
| 8 | -1.39528 | -0.13894 | 0.019633 | 1.024586 | 0.043412 | 5526.211 | 0.009199 | 0.919872 |  |
| 9 | 0.804684 | 0.074007 | 0.00547 | 1.007092 | 0.042625 | 5653.316 | 0.008297 | 0.82969 |  |
| 10 | 0.501884 | 0.043133 | 0.00186 | 1.007385 | 0.042685 | 5666.603 | 0.007332 | 0.733194 |  |
| 11 | 0.43642 | 0.042014 | 0.001765 | 1.009052 | 0.042676 | 5551.676 | 0.009156 | 0.915635 |  |
| 12 | 1.905416 | 0.168328 | 0.028217 | 1.003159 | 0.042471 | 5637.804 | 0.007618 | 0.761802 |  |
| 13 | 1.217685 | 0.110694 | 0.012221 | 1.005477 | 0.042559 | 5646.822 | 0.008073 | 0.807301 |  |
| 14 | 0.882095 | 0.079056 | 0.006243 | 1.006864 | 0.042633 | 5657.58 | 0.007899 | 0.789944 |  |
| 15 | 0.030595 | 0.00084 | 7.11E-07 | 1.015821 | 0.043001 | 5648.161 | 0.009084 | 0.908376 |  |
| 16 | 0.019397 | 0.000821 | 6.76E-07 | 1.012215 | 0.042839 | 5661.356 | 0.008907 | 0.890713 |  |
| 17 | -0.8395 | -0.08857 | 0.008072 | 1.035503 | 0.043932 | 5662.393 | 0.009181 | 0.918076 |  |
| 18 | -0.57568 | -0.0546 | 0.00299 | 1.011453 | 0.042815 | 5671.83 | 0.008642 | 0.864151 |  |
| 19 | 3.473913 | 0.355736 | 0.117248 | 0.940547 | 0.039437 | 5219.912 | 0.008925 | 0.892464 | * |
| 20 | 1.75826 | 0.152808 | 0.02328 | 1.004065 | 0.042524 | 5645.523 | 0.007397 | 0.739695 |  |
| 21 | -1.22324 | -0.11418 | 0.013059 | 1.010168 | 0.042758 | 5666.264 | 0.008567 | 0.856697 |  |
| 22 | -1.24201 | -0.11929 | 0.014286 | 1.012788 | 0.042863 | 5657.763 | 0.008968 | 0.896763 |  |
| 23 | 0.289949 | 0.026795 | 0.000719 | 1.009505 | 0.042723 | 5660.297 | 0.008628 | 0.862795 |  |
| 24 | 0.13702 | 0.01234 | 0.000153 | 1.010231 | 0.042756 | 5663.402 | 0.008667 | 0.86669 |  |
| 25 | -0.48594 | -0.04428 | 0.001963 | 1.009525 | 0.042751 | 5671.78 | 0.008073 | 0.807301 |  |
| 26 | -0.03975 | -0.00335 | 1.12E-05 | 1.006943 | 0.042707 | 5671.115 | 0.006445 | 0.644456 |  |
| 27 | 1.098368 | 0.094779 | 0.008973 | 1.00614 | 0.042626 | 5658.919 | 0.007332 | 0.733194 |  |
| 28 | 4.006344 | 0.400155 | 0.149708 | 0.947294 | 0.039764 | 5271.377 | 0.008783 | 0.878321 | * |
| 29 | -1.67538 | -0.15155 | 0.022962 | 1.008018 | 0.042677 | 5661.807 | 0.008121 | 0.812129 |  |
| 30 | -0.66901 | -0.0652 | 0.004275 | 1.014413 | 0.042941 | 5671.67 | 0.008937 | 0.893703 |  |
| 31 | 1.19822 | 0.11546 | 0.013226 | 1.001472 | 0.042333 | 5602.235 | 0.008819 | 0.881883 |  |
| 32 | -1.52746 | -0.13342 | 0.017804 | 1.007744 | 0.042691 | 5666.585 | 0.007568 | 0.756787 |  |
| 33 | -1.62316 | -0.14513 | 0.021061 | 1.00796 | 0.042684 | 5663.948 | 0.007932 | 0.793186 |  |
| 34 | -0.27552 | -0.02143 | 0.000459 | 1.006429 | 0.042707 | 5671.709 | 0.005932 | 0.593177 |  |
| 35 | 0.340012 | 0.029007 | 0.000842 | 1.007552 | 0.042696 | 5668.253 | 0.007262 | 0.726218 |  |
| 36 | 3.242803 | 0.262976 | 0.068787 | 0.999612 | 0.042359 | 5625.863 | 0.006445 | 0.644456 |  |
| 37 | 3.651258 | 0.312058 | 0.096435 | 0.995816 | 0.042147 | 5597.198 | 0.007106 | 0.710614 | * |
| 38 | -1.17683 | -0.09246 | 0.008551 | 1.006449 | 0.042699 | 5670.458 | 0.006122 | 0.61224 |  |
| 39 | 0.623274 | 0.053088 | 0.002818 | 1.007052 | 0.042676 | 5665.83 | 0.007187 | 0.718712 |  |
| 40 | 1.242275 | 0.108656 | 0.011787 | 1.005751 | 0.042599 | 5654.817 | 0.007515 | 0.751453 |  |
| 41 | 4.574242 | 0.405894 | 0.161453 | 0.985659 | 0.041642 | 5530.389 | 0.007568 | 0.756787 | * |
| 42 | 0.541787 | 0.050766 | 0.002576 | 1.008279 | 0.042664 | 5651.834 | 0.008654 | 0.865448 |  |
| 43 | -1.23668 | -0.12397 | 0.015652 | 1.025958 | 0.043478 | 5608.313 | 0.00918 | 0.917961 |  |
| 44 | -0.01492 | -0.00208 | 4.32E-06 | 1.010931 | 0.042787 | 5666.146 | 0.008707 | 0.870651 |  |
| 45 | -0.72625 | -0.0941 | 0.009728 | 1.097916 | 0.046897 | 5659.483 | 0.009222 | 0.922195 |  |
| 46 | 1.184544 | 0.112172 | 0.012517 | 1.003712 | 0.042448 | 5624.407 | 0.008635 | 0.863481 |  |
| 47 | 1.857978 | 0.16077 | 0.025764 | 1.003759 | 0.042513 | 5644.228 | 0.007332 | 0.733194 |  |
| 48 | 0.310512 | 0.027339 | 0.000748 | 1.008152 | 0.042701 | 5667.2 | 0.007752 | 0.775189 |  |
| 49 | -1.91791 | -0.18065 | 0.032544 | 1.006345 | 0.042562 | 5628.515 | 0.008871 | 0.887069 |  |
| 50 | -0.67705 | -0.06615 | 0.004403 | 1.014823 | 0.04296 | 5671.621 | 0.008958 | 0.895765 |  |
| 51 | 2.298102 | 0.240484 | 0.054275 | 0.952686 | 0.040006 | 5272.231 | 0.009056 | 0.905598 |  |
| 52 | -0.72981 | -0.09518 | 0.009983 | 1.10102 | 0.047045 | 5658.157 | 0.009223 | 0.922261 |  |
| 53 | -0.67756 | -0.12166 | 0.019052 | 1.266977 | 0.054951 | 5617.35 | 0.009235 | 0.923494 |  |
| 54 | -0.6627 | -0.06998 | 0.00501 | 1.030088 | 0.043675 | 5671.342 | 0.009161 | 0.916127 |  |
| 55 | -0.96029 | -0.09537 | 0.009204 | 1.020122 | 0.043204 | 5662.683 | 0.009108 | 0.910761 |  |
| 56 | -1.17683 | -0.09246 | 0.008551 | 1.006449 | 0.042699 | 5670.458 | 0.006122 | 0.61224 |  |
| 57 | -0.39926 | -0.03541 | 0.001255 | 1.008732 | 0.042733 | 5671.695 | 0.007665 | 0.766524 |  |
| 58 | -0.4654 | -0.04235 | 0.001796 | 1.009465 | 0.042749 | 5671.753 | 0.008047 | 0.804727 |  |
| 59 | 0.504701 | 0.044952 | 0.002021 | 1.007882 | 0.042683 | 5664.262 | 0.007865 | 0.786536 |  |
| 60 | -1.03901 | -0.09259 | 0.00858 | 1.0088 | 0.042728 | 5670.332 | 0.00783 | 0.782951 |  |
| 61 | 2.657154 | 0.2198 | 0.048107 | 1.001491 | 0.042435 | 5635.453 | 0.006706 | 0.670639 |  |
| 62 | -0.96049 | -0.08769 | 0.007699 | 1.009588 | 0.042749 | 5670.513 | 0.008187 | 0.818667 |  |
| 63 | 1.036893 | 0.096008 | 0.009194 | 1.005886 | 0.042565 | 5644.752 | 0.008358 | 0.835842 |  |
| 64 | -1.13213 | -0.08755 | 0.007667 | 1.006261 | 0.042699 | 5670.673 | 0.005932 | 0.593177 |  |
| 65 | -1.05484 | -0.1033 | 0.010762 | 1.017001 | 0.043058 | 5660.486 | 0.00907 | 0.907047 |  |
| 66 | -1.99837 | -0.18897 | 0.035457 | 1.002618 | 0.042376 | 5580.126 | 0.009046 | 0.904604 |  |
| 67 | 0.397994 | 0.037766 | 0.001427 | 1.009788 | 0.042718 | 5628.942 | 0.009026 | 0.902601 |  |
| 68 | 0.777733 | 0.067587 | 0.004566 | 1.006961 | 0.042659 | 5662.821 | 0.007458 | 0.745768 |  |
| 69 | 1.512936 | 0.148107 | 0.0216 | 0.994699 | 0.042008 | 5554.983 | 0.008894 | 0.889434 |  |
| 70 | 0.47231 | 0.038791 | 0.001505 | 1.006811 | 0.042688 | 5668.285 | 0.006706 | 0.670639 |  |
| 71 | 2.730402 | 0.272123 | 0.070986 | 0.970175 | 0.040845 | 5406.374 | 0.008871 | 0.887069 | * |
| 72 | -1.02634 | -0.09834 | 0.00971 | 1.012777 | 0.042866 | 5667.022 | 0.008883 | 0.88829 |  |
| 73 | -1.79168 | -0.1631 | 0.025676 | 0.977987 | 0.041199 | 5031.158 | 0.00922 | 0.921994 |  |
| 74 | -1.58098 | -0.15301 | 0.023483 | 1.012017 | 0.042816 | 5520.401 | 0.009181 | 0.918121 |  |
| 75 | 0.620474 | 0.058618 | 0.003432 | 1.007719 | 0.042633 | 5644.857 | 0.008741 | 0.874101 |  |
| 76 | -0.87379 | -0.0791 | 0.006264 | 1.009359 | 0.042744 | 5671.105 | 0.008047 | 0.804727 |  |
| 77 | -0.27139 | -0.02575 | 0.000664 | 1.01056 | 0.04278 | 5670.646 | 0.008493 | 0.849251 |  |
| 78 | 0.841774 | 0.07044 | 0.00496 | 1.006466 | 0.042661 | 5664.427 | 0.006923 | 0.692338 |  |
| 79 | -1.31512 | -0.10815 | 0.011699 | 1.007011 | 0.042698 | 5669.524 | 0.006706 | 0.670639 |  |
| 80 | -1.66875 | -0.15073 | 0.022716 | 1.008014 | 0.042678 | 5662.117 | 0.008098 | 0.809766 |  |
| 81 | 0.129258 | 0.011603 | 0.000135 | 1.009943 | 0.042747 | 5664.875 | 0.008567 | 0.856697 |  |
| 82 | -0.42494 | -0.04013 | 0.001614 | 1.010862 | 0.042792 | 5671.507 | 0.008532 | 0.853169 |  |
| 83 | 1.462945 | 0.125107 | 0.015623 | 1.005136 | 0.042585 | 5654.003 | 0.007187 | 0.718712 |  |
| 84 | -1.84672 | -0.1724 | 0.029682 | 1.007479 | 0.042625 | 5646.013 | 0.008679 | 0.867881 |  |
| 85 | -0.40077 | -0.03435 | 0.001181 | 1.008012 | 0.042722 | 5671.751 | 0.007187 | 0.718712 |  |
| 86 | 1.850915 | 0.145863 | 0.021243 | 1.004051 | 0.042585 | 5655.653 | 0.006122 | 0.61224 |  |
| 87 | -0.20943 | -0.02143 | 0.000462 | 1.013919 | 0.042917 | 5667.055 | 0.00895 | 0.895006 |  |
| 88 | -0.10547 | -0.00918 | 8.43E-05 | 1.007891 | 0.042716 | 5671.006 | 0.007187 | 0.718712 |  |
| 89 | -0.59487 | -0.05525 | 0.003059 | 1.010325 | 0.042775 | 5671.829 | 0.008373 | 0.837256 |  |
| 90 | 0.599537 | 0.046328 | 0.002146 | 1.005918 | 0.042683 | 5668.559 | 0.005932 | 0.593177 |  |
| 91 | 2.711036 | 0.268997 | 0.069566 | 0.972685 | 0.040965 | 5423.345 | 0.008849 | 0.884931 |  |
| 92 | 2.275977 | 0.199825 | 0.039717 | 1.001667 | 0.042405 | 5629.625 | 0.007515 | 0.751453 |  |
| 93 | -1.85068 | -0.17287 | 0.029842 | 1.00744 | 0.042622 | 5645.328 | 0.00869 | 0.869023 |  |
| 94 | -1.10202 | -0.1073 | 0.011596 | 1.015707 | 0.042998 | 5659.629 | 0.009048 | 0.904811 |  |
| 95 | -0.27552 | -0.02143 | 0.000459 | 1.006429 | 0.042707 | 5671.709 | 0.005932 | 0.593177 |  |
| 96 | -0.46693 | -0.03779 | 0.001429 | 1.007058 | 0.042712 | 5671.827 | 0.006445 | 0.644456 |  |
| 97 | 0.10387 | 0.008884 | 7.90E-05 | 1.008214 | 0.042713 | 5669.489 | 0.007568 | 0.756787 |  |
| 98 | 1.132849 | 0.112356 | 0.012448 | 0.996266 | 0.042076 | 5547.48 | 0.00902 | 0.901989 |  |
| 99 | -0.26882 | -0.02357 | 0.000556 | 1.008358 | 0.042725 | 5671.438 | 0.007458 | 0.745768 |  |
| 100 | -1.40388 | -0.13263 | 0.017616 | 1.010312 | 0.042754 | 5658.46 | 0.008787 | 0.878704 |  |
| 101 | -0.16397 | -0.01365 | 0.000186 | 1.007312 | 0.042712 | 5671.38 | 0.006706 | 0.670639 |  |
| 102 | 3.813183 | 0.37676 | 0.134268 | 0.957334 | 0.040242 | 5335.27 | 0.008736 | 0.873636 | * |
| 103 | -1.91993 | -0.18088 | 0.032624 | 1.006295 | 0.042559 | 5627.802 | 0.008876 | 0.887567 |  |
| 104 | -0.34594 | -0.02731 | 0.000746 | 1.006661 | 0.042709 | 5671.768 | 0.006122 | 0.61224 |  |
| 105 | 0.937254 | 0.092485 | 0.008469 | 1.000006 | 0.042253 | 5567.546 | 0.009033 | 0.90326 |  |
| 106 | 0.495947 | 0.047393 | 0.002245 | 1.008474 | 0.042657 | 5629.746 | 0.008978 | 0.89782 |  |
| 107 | 0.250086 | 0.022247 | 0.000495 | 1.008528 | 0.042709 | 5667.073 | 0.007963 | 0.796276 |  |
| 108 | -0.27071 | -0.02529 | 0.000641 | 1.009994 | 0.042762 | 5670.91 | 0.008313 | 0.831307 |  |
| 109 | 1.009256 | 0.092321 | 0.008507 | 1.006275 | 0.042591 | 5650.093 | 0.008187 | 0.818667 |  |
| 110 | -0.02147 | -0.00493 | 2.46E-05 | 1.018402 | 0.043122 | 5646.372 | 0.009117 | 0.911701 |  |
| 111 | -0.50812 | -0.05109 | 0.002633 | 1.017388 | 0.043078 | 5671.446 | 0.009042 | 0.904249 |  |
| 112 | -0.72987 | -0.07014 | 0.004941 | 1.012892 | 0.042874 | 5671.406 | 0.008835 | 0.883464 |  |
| 113 | -1.90245 | -0.1789 | 0.031931 | 1.00669 | 0.04258 | 5633.483 | 0.008832 | 0.883157 |  |
| 114 | -1.45039 | -0.12411 | 0.015407 | 1.007506 | 0.042694 | 5667.971 | 0.007262 | 0.726218 |  |
| 115 | -1.67538 | -0.15155 | 0.022962 | 1.008018 | 0.042677 | 5661.807 | 0.008121 | 0.812129 |  |
| 116 | -1.86529 | -0.17459 | 0.030434 | 1.007277 | 0.042613 | 5642.573 | 0.008732 | 0.873162 |  |
| 117 | -0.83313 | -0.08827 | 0.008027 | 1.036576 | 0.043982 | 5662.51 | 0.009183 | 0.918292 |  |
| 118 | 4.386977 | 0.377824 | 0.140707 | 0.990694 | 0.0419 | 5564.913 | 0.007187 | 0.718712 | * |
| 119 | -0.94329 | -0.08292 | 0.006882 | 1.008535 | 0.042726 | 5670.962 | 0.007618 | 0.761802 |  |
| 120 | -0.34642 | -0.03514 | 0.001244 | 1.015869 | 0.043007 | 5669.263 | 0.009011 | 0.901149 |  |
| 121 | -1.43181 | -0.12189 | 0.01486 | 1.007443 | 0.042695 | 5668.24 | 0.007187 | 0.718712 |  |
| 122 | -0.78581 | -0.07049 | 0.004974 | 1.009119 | 0.04274 | 5671.487 | 0.007899 | 0.789944 |  |
| 123 | -1.00337 | -0.08897 | 0.007923 | 1.008703 | 0.042728 | 5670.598 | 0.007752 | 0.775189 |  |

## **Influence of Study-Level Quality Factors**

### **MEDQUARG score**

### **MEDQUARG score greater than or equal to 6**

### **Publication year**

### **Number of samples tested.**

### **Therapeutic category**

### **Subregion**

### **Random sampling approach**

### **Mystery shopper approach**
