# Supplementary material for: Nearly one in five essential medicines fail quality standards in Africa: A systematic review and meta-analysis of MNCH medicines and antimalarials
Source: PLOS Glob Public Health. 2026 Sep 2;6(9):e0006326. doi: 10.1371/journal.pgph.0006326 (PMC13537570; doi:10.1371/journal.pgph.0006326)
Supplement: Supplementary file 10 [file pgph.0006326.s010.docx]

# **S5 Table. Studies Excluded from Meta-analysis**

***Reason****: Studies collected and tested less than 10 samples.*

| **#** | **Source** | **Country** | **Therapeutic Category** | **Total Samples** | **MEDQUARG Score** | **Sampling Method** | **Purchasing Method** | **Tested Other Ingredients** |
| --- | --- | --- | --- | --- | --- | --- | --- | --- |
| 1 | Alhedethe et al., 2017 | Sierra Leone | Antibiotic | 6 | 2 | Convenience | Overt | Yes |
| 2 | Ali et al., 2011 | Nigeria | Antimalarial | 6 | 3 | Convenience | Overt | No |
| 3 | Cáceres-Pérez et al., 2024 | Mauritania | Antimalarial, Antiretroviral | 5 | 6 | Targeted/Risk-based | Overt | No |
| 4 | Chaccour et al., 2012 | Equatorial Guinea | Antimalarial | 1 | 7 | Targeted/Risk-based | Overt | No |
| 5 | Chigozie et al., 2014 | Nigeria | Vaccine | 3 | 4 | Convenience | Overt | No |
| 6 | Chiumia et al., 2025 | Malawi | Antibiotic | 9 | 8 | Targeted | Overt | No |
| 7 | Desta et al., 2025 | Ethiopia | Antibiotic | 5 | 5 | Random | N/S | Yes |
| 8 | Gnegel et al., 2020 | Cameroon, DR Congo | Antimalarial | 5 | 5 | Targeted/Risk-based | N/S | Yes |
| 9 | Hagen et al., 2020 | Malawi, Rwanda | Uterotonic | 4 | 7 | Targeted/Risk-based | Overt, Mystery client | Yes |
| 10 | Haruna et al., 2013 | Nigeria | Antihypertensive | 4 | 3 | Convenience | Overt | No |
| 11 | Hasen et al., 2024 | Ethiopia | Antimalarial | 6 | 3 | N/S | Overt | Yes |
| 12 | Kassahun et al., 2024 | Ethiopia | Antibiotic | 7 | 5 | Convenience | Overt | Yes |
| 13 | Mekasha et al., 2023 | Ethiopia | Antibiotic | 6 | 8 | Convenience | Mystery client | No |
| 14 | Mustapha et al., 2022 | Nigeria | Antibiotic | 6 | 4 | Random | Overt | Yes |
| 15 | Newton et al., 2014 | Angola | Antimalarial | 1 | 7 | Targeted/Risk-based | Seized | Yes |
| 16 | Nyarko et al., 2013 | Ghana | Antimalarial | 9 | 6 | Random | Overt | No |
| 17 | Ogochukwu et al., 2018 | Nigeria | Antibiotic | 4 | 6 | Random | Mystery client | Yes |
| 18 | Oli et al., 2020 | Nigeria | Antibiotic | 8 | 6 | Convenience | Overt | Yes |
| 19 | Pichini et al., 2015 | Senegal | Analgesic | 5 | 5 | Convenience | Overt | Yes |
| 20 | Tchounga et al., 2021 | Cameroon, DR Congo, Niger | Antimalarial | 8 | 6 | Targeted/Risk-based | N/S | Yes |

***API:** Active pharmaceutical ingredient; **DART-MS:** Direct analysis in real time–mass spectrometry; **DART-MS/MS:** Direct analysis in real time–tandem mass spectrometry; **GPHF:** Global Pharma Health Fund; **HPLC:** High-performance liquid chromatography; **HPLC-DAD:** High-performance liquid chromatography–diode array detection; **HPLC-UV:** High-performance liquid chromatography with ultraviolet detection; **HPTLC:** High-performance thin-layer chromatography; **NMR:** Nuclear magnetic resonance; **UHPLC-MS:** Ultra-high-performance liquid chromatography–mass spectrometry; **UHPLC-TWIMS-MS:** Ultra-high-performance liquid chromatography–traveling wave ion mobility spectrometry–mass spectrometry;

**Bibliography of Studies Excluded from the Meta-analysis**

1. Alhedethe A, Alhudaithy K, Zloh AM. An Evaluation of Prevalence of Low Quality of Medicines in Saudi Arabia and Factors Associated an Analytical Comparative Study. Arch Chem Res. 2017;2(1):1.
2. Ali SA, Ezika CC, Abdulraheem OR, Alemika TE, Sule IM, Ilyas M, et al. Quality Assessment of Artemisinin-Based Drugs Marketed and Used In Maiduguri Metropolitan Council Borno State, Nigeria. Research Journal of Pharmaceutical, Biological and Chemical Sciences. 2016;7(3):2844–52.
3. Cáceres-Pérez AR, Suárez-González J, Santoveña-Estévez A, Fariña JB. Quality assessment of oral antimalarial and antiretroviral medicines used by public health systems in Sahel countries. PLoS ONE. 2024;19(5): e0303289.
4. Chaccour CJ, Kaur H, Mabey D, Del Pozo JL. Travel and fake artesunate: a risky business. The Lancet. 2012;380(9850):1120.
5. Chigozie, V. U., P. M. Eze, C. R. Chukwunwejim, C. C. Abba, C. S. Nworu, and C. O. Esimone. Quality Assessment of Some Brands of Tetanus Toxoids Marketed in Open Markets in South-Eastern Nigeria. Journal of Pharmaceutical Research International. 2015;4(13):1657-67.
6. Chiumia FK, Sinjani Muula A, Chimimba F, Nyirongo HM, Kampira E and Khuluza F. Substandard antibiotics and their clinical outcomes among hospitalized patients in southern Malawi: a pilot study. Front. Pharmacol. 2025;16:1535501.
7. Desta HK, Ketema G, Van Schepdael A, Adams E. A Cost-Effective Liquid Chromatography Method with Ultraviolet Detection for Identity Screening and Assay of Injectable Antibiotics. Molecules. 2025; 30(10):2151.
8. Gnegel G, Hauk C, Neci R, Mutombo G, Nyaah F, Wistuba D, et al. Identification of Falsified Chloroquine Tablets in Africa at the Time of the COVID-19 Pandemic. Am J Trop Med Hyg. 2020;103(1):73–6.
9. Hagen N, Bizimana T, Kayumba PC, Khuluza F, Heide L (2020) Stability of misoprostol tablets collected in Malawi and Rwanda: Importance of intact primary packaging. PLoS ONE 15(9): e0238628.
10. Haruna B, Adaku OE, Usifoh CO. Chemical equivalence study on four brands of α-methyldopa. Journal of Chemical and Pharmaceutical Research. 2013;5(10):356–8. Accessed from: <https://www.jocpr.com/articles/chemical-equivalence-study-on-four-brands-of-methyldopa.pdf>
11. Hasen, G., Birhane, W., Suleman, S., & Ashenef, A. Development and validation of a quantitative minilab system for quality evaluation of selected medicines: Albendazole, arthemether-lumefantrine combination (Co-artem®), and mebendazole finished pharmaceutical products. Acta Chromatographica. 2025;37(1), 14-25.
12. Kassahun H, Van Schepdael A, Ketema G, Adams E. Development and validation of a simple and affordable LC-UV method for identification and assay of selected antimicrobial medicines. Journal of Pharmaceutical and Biomedical Analysis. 2024;244:116127.
13. Mekasha, Y. T., Chali, B. U., Feissa, A. B., Godena, G. H., Hassen, H. K., Wega, S. S. Quality evaluation of the Azithromycin tablets commonly marketed in Adama, and Modjo towns, Oromia Regional State, Ethiopia. PloS one. 2023;18(3), e0282156.
14. Mustapha, K.B., Kirim, R.A., Galadima, I.H., Odeniran, O.A., Aboh, M.I., Oladosu, P.O. Comparative quality assessment of six commercial brands of metronidazole infusions sold in Abuja, Nigeria. Journal of Phytomedicine and Therapeutics. 2022;21(2), 786–799.
15. Newton PN, Tabernero P, Dwivedi P, Culzoni MJ, Monge ME, Swamidoss I, et al. Falsified medicines in Africa: all talk, no action. The Lancet Global Health. 2014;2(9):e509–e10.
16. Nyarko, E. and Nettey, H. Quality Assessment of Artemether/Lumefantrine Tablets Sampled from Pharmacies in Accra, Using the MVHimagePCv8.exe Color Software. Pharmacology & Pharmacy. 2013;4,567-572.
17. Ogochukwu U, Christianah I, Marlene E, Sabinus O, Martins E. Quality Assessment of Some Brands of Clarithromycin and Azithromycin Tablets Using the Concept of Dissolution Efficiency and Similarity Factor. International Journal of Pharmaceutical Sciences and Research. 2018;9(12):5401–10.
18. Oli AN, Ibeabuchi MU, Enweani IB, Emencheta SC. Pharmaceutical Quality of Selected Metronidazole and Ciprofloxacin Infusions Marketed in South Eastern Nigeria. Drug, Healthcare and Patient Safety. 2020;12:95–106.
19. Pichini S, Rotolo MC, Bellotti P, Minutillo A, Mastrobattista L, Pacifici R. Quali-quantitative analysis of best selling drugs from pharmacy, street market and traditional herbal medicine: A pilot study of market surveillance in Senegal. Journal of Pharmaceutical and Biomedical Analysis. 2015;104:62–6.
20. Tchounga CA, Sacré PY, Ciza P, Ngono R, Ziemons E, Hubert Ph, et al. Composition analysis of falsified chloroquine phosphate samples seized during the COVID-19 pandemic. Journal of Pharmaceutical and Biomedical Analysis. 2021;194:113761.
