# Supplementary material for: Nearly one in five essential medicines fail quality standards in Africa: A systematic review and meta-analysis of MNCH medicines and antimalarials
Source: PLOS Glob Public Health. 2026 Sep 2;6(9):e0006326. doi: 10.1371/journal.pgph.0006326 (PMC13537570; doi:10.1371/journal.pgph.0006326)
Supplement: Supplementary file 11 [file pgph.0006326.s012.docx]

**Response to Reviewers**

We thank the editorial team and reviewers for their careful evaluation of the manuscript and thoughtful suggestions that have helped us to improve the manuscript substantially. We have responded to each suggestion and question raised by the editorial team and reviewers in the text below. We hope our responses are satisfactory.

**Checklist**

|  | **Include these items when submitting the revised manuscript** | **Yes** | **No** |
| --- | --- | --- | --- |
| **1** | A letter that responds to each point raised by the editor and reviewer(s). Upload this letter as a separate file labeled ‘Response to Reviewers’. | X |  |
| **2** | A marked-up copy of the manuscript that highlights changes made to the original version. Upload this as a separate file labeled ‘Revised Manuscript with Track Changes’. | X |  |
| **3** | An unmarked version of the revised paper without tracked changes. Upload this a separate file labeled ‘Manuscript’. | X |  |

**Response to editor’s comments:**

1. We have noticed that you have uploaded Supporting Information files, but you have not included a list of legends. Please add a full list of legends for your Supporting Information files after the references list.

Response: Thank you for noting this omission. We have added a complete list of legends for all Supporting Information files after the reference list on page 31 of the revised manuscript.

2. As required by our policy on Data Availability, please ensure your manuscript or supplementary information includes the following:
   a. A numbered table of all studies identified in the literature search, including those that were excluded from the analyses. For every excluded study, the table should list the reason(s) for exclusion.

Response: Thank you. A numbered table of all studies included in the systematic review and meta-analysis is provided in S4 Appendix. A numbered table of the studies excluded after full-text assessment, together with the specific reason or reasons for exclusion, is provided in S10 Appendix.

b. If any of the included studies are unpublished, include a link (URL) to the primary source or detailed information about how the content can be accessed.

Response: Not applicable. No unpublished studies were included in the analysis. All included studies and reports are published and publicly available. For sources that may be difficult to locate, we have added direct URLs to the reference list to facilitate access.

c. A table of all data extracted from the primary research sources for the systematic review and/or meta-analysis. The table must include the following information for each study:
   i. Name of data extractors and date of data extraction
   ii. Confirmation that the study was eligible to be included in the review.
   iii. All data extracted from each study for the reported systematic review and/or meta-analysis that would be needed to replicate your analyses.
   iv. If data or supporting information were obtained from another source (e.g. correspondence with the author of the original research article), please provide the source of data and dates on which the data/information were obtained by your research group.

Response: A complete study-level data extraction table has been provided as S11 Appendix. The table includes the names of the data extractors, dates of data extraction, confirmation that each study met the eligibility criteria, and the study-level data used to replicate the systematic review and meta-analysis. All data and supporting information were obtained directly from the included primary research reports and their associated supplementary materials; no information was obtained through author correspondence or from external sources. Therefore, item iv is not applicable.

d. If applicable for your analysis, a table showing the completed risk of bias and quality/certainty assessments for each study or outcome. Please ensure this is provided for each domain or parameter assessed. For example, if you used the Cochrane risk-of-bias tool for randomized trials, provide answers to each of the signalling questions for each study.

Response: This request is applicable to the methodological quality assessment conducted in our review. We assessed for bias and quality of the studies using the 12-item MEDQUARG checklist. We have revised S3 Appendix to provide the item-level judgment or score for each MEDQUARG criterion for every included study, together with the overall MEDQUARG score. The definitions and scoring criteria for the individual MEDQUARG items are described in S2 Appendix.

e. If you used GRADE to assess certainty of evidence, provide judgements about each of the quality of evidence factor. This should be provided for each outcome.

Response: Not applicable. We did not use GRADE to assess certainty of evidence. Methodological quality was assessed at the study level using the 12-item MEDQUARG checklist.

f. An explanation of how missing data were handled. This information can be included in the main text, supplementary information, or relevant data repository.

Response: Thank you. We added a statement to the Methods under “Data Extraction and Definitions” explaining how missing or incompletely reported information was handled. When samples were reported only as “poor quality” without distinguishing between substandard and falsified products, they were classified as substandard unless the original investigators explicitly identified them as counterfeit or falsified. When sample-level API content or other testing details were unavailable, these values were not imputed; classifications were based on the aggregate findings and terminology reported in the original study. Where only aggregate regional or category-level data were reported, the Methods now also explain how these counts were proportionally allocated for subgroup analyses.

g. Please note that providing these underlying data is a requirement for publication in this journal, and if these data are not provided your manuscript might be rejected.

Response: We acknowledge this requirement. All underlying data needed to reproduce the reported analyses are now provided in the Supporting Information. The complete study-level data extraction dataset and analysis variables are included in S11 Appendix. The full search strategies are provided in S1 Appendix, the completed MEDQUARG assessments are provided in S3 Appendix, and the study-level prevalence estimates are provided in S5 Appendix.

3. We note that your Data Availability Statement is currently as follows: "All data underlying this systematic review and meta-analysis are available within the manuscript and its supplementary appendices, including the full search strategies (S1 Appendix), MEDQUARG quality assessment scores (S3 Appendix), and study-level prevalence estimates (S5 Appendix). The dataset used for meta-analysis, including extracted study-level data, effect sizes, and meta-regression variables, will be made available upon reasonable request to the Johns Hopkins BESAFE program at besafe@jh.edu."
   a. Please confirm at this time whether or not your submission contains all raw data required to replicate the results of your study. Authors must share the “minimal data set” for their submission. PLOS defines the minimal data set to consist of the data required to replicate all study findings reported in the article, as well as related metadata and methods (https://journals.plos.org/plosone/s/data-availability#loc-minimal-data-set-definition). For example, authors should submit the following data:
      i. The values behind the means, standard deviations and other measures reported
      ii. The values used to build graphs
      iii. The points extracted from images for analysis. Authors do not need to submit their entire data set if only a portion of the data was used in the reported study.

Response: We confirm that the revised submission includes the minimal dataset required to replicate the reported findings. S11 Appendix contains the extracted study-level data and analysis variables underlying the pooled estimates, subgroup analyses, meta-regression, tables, and figures.

b. If your submission does not contain these data, please either upload them as Supporting Information files or deposit them to a stable, public repository and provide us with the relevant URLs, DOIs, or accession numbers. For a list of recommended repositories, please see <https://journals.plos.org/plosone/s/recommended-repositories>.

Response: The complete dataset required to reproduce the analyses has been uploaded directly with the submission as S11 Appendix. Therefore, depositing in an external repository is not needed.

c. If there are ethical or legal restrictions on sharing a de-identified data set, please explain them in detail (e.g., data contain potentially sensitive information, data are owned by a third-party organization, etc.) and who has imposed them (e.g., an ethics committee).

Response: All data were extracted from publicly available studies and their associated supplementary materials.

d. Please also provide contact information for a data access committee, ethics committee, or other institutional body to which data requests may be sent.

Response: The dataset is fully available in S11 Appendix and is not subject to restricted-access procedures. Therefore, no data access committee, ethics committee, or other institutional body is required to review data requests.

e. If data are owned by a third party, please indicate how others may request data access.

Response: The dataset is not owned or controlled by a third party. All extracted data underlying the analyses are provided in S11 Appendix and may be accessed directly through the Supporting Information.

4. Some material included in your submission may be copyrighted. According to PLOS’s copyright policy, authors who use figures or other material (e.g., graphics, clipart, maps) from another author or copyright holder must demonstrate or obtain permission to publish this material under the Creative Commons Attribution 4.0 International (CC BY 4.0) License used by PLOS journals. Please closely review the details of PLOS’s copyright requirements here: PLOS Licenses and Copyright. If you need to request permissions from a copyright holder, you may use PLOS's Copyright Content Permission form. Please respond directly to this email or email the journal office and provide any known details concerning your material's license terms and permissions required for reuse, even if you have not yet obtained copyright permissions or are unsure of your material's copyright compatibility.
   a. Potential Copyright Issues: Figure 2: please
      i. (a) provide a direct link to the base layer of the map (i.e., the country or region border shape) and ensure this is also included in the figure legend; and
      ii. (b) provide a link to the terms of use / license information for the base layer image or shapefile.

We cannot publish proprietary or copyrighted maps (e.g. Google Maps, Mapquest) and the terms of use for your map base layer must be compatible with our CC-BY 4.0 license. Note: if you created the map in a software program like R or ArcGIS, please locate and indicate the source of the basemap shapefile onto which data has been plotted. If your map was obtained from a copyrighted source please amend the figure so that the base map used is from an openly available source. Alternatively, please provide explicit written permission from the copyright holder granting you the right to publish the material under our CC-BY 4.0 license. Please note that the following CC BY licenses are compatible with PLOS license: CC BY 4.0, CC BY 2.0 and CC BY 3.0, meanwhile such licenses as CC BY-ND 3.0 and others are not compatible due to additional restrictions. If you are unsure whether you can use a map or not, please do reach out and we will be able to help you. The following websites are good examples of where you can source open access or public domain maps: * U.S. Geological Survey (USGS) - All maps are in the public domain. (http://www.usgs.gov) * PlaniGlobe - All maps are published under a Creative Commons license so please cite “PlaniGlobe, http://www.planiglobe.com, CC BY 2.0” in the image credit after the caption. (http://www.planiglobe.com/?lang=enl) * Natural Earth - All maps are public domain. (http://www.naturalearthdata.com/about/terms-of-use/)

Response: Thank you for your guidance regarding Figure 2 and the CC BY 4.0 requirement. We have removed the original Excel/Bing Maps version of Figure 2 and recreated it entirely in R using Natural Earth's public-domain 1:50m country boundary dataset, which appears on your list of recommended open-source sources. Study counts were joined by ISO country code, and we confirmed the values match the underlying extraction data. The high-resolution TIFF has been uploaded, and the Figure 2 caption now includes full attribution and license information. Figure 2 is derived solely from open-source data compatible with PLOS's CC BY 4.0 license.

Dataset: Natural Earth 1:50m Admin 0 Countries (public domain). <https://www.naturalearthdata.com/downloads/50m-cultural-vectors/50m-admin-0-countries-2/>
Terms of use: <https://www.naturalearthdata.com/about/terms-of-use/>

5. If the reviewer comments include a recommendation to cite specific previously published works, please review and evaluate these publications to determine whether they are relevant and should be cited. There is no requirement to cite these works unless the editor has indicated otherwise.
6. **Additional Editor Comments** (if provided): The first reviewer provided this additional feedback:
   a. The information presented is critical for informing policy and strengthening the body of knowledge on SFMP prevention. However, the manuscript requires substantial review to align with international reporting guidelines. In particular,
      i. issues related to publication bias should be explicitly addressed.

Response: Thank you for this important comment. We have explicitly addressed publication bias in the revised manuscript. Publication bias was evaluated using funnel plots and Egger’s regression test, and the results are reported in the Methods, Results, and Discussion, with additional diagnostic analyses provided in S9 Appendix. The analyses identified funnel-plot asymmetry and statistically significant small-study effects, which are now clearly acknowledged when interpreting the pooled estimates.

ii. Additionally, subgroup meta-analyses based on quality control parameters should be conducted and clearly reported.

Response: Thank you for this suggestion. We conducted and clearly reported subgroup and meta-regression analyses examining relevant study-level methodological and quality-related factors. These included therapeutic category, geographic subregion, sampling method, mystery-client purchasing, publication year, sample size, and MEDQUARG score assessed both continuously and using the ≥6 threshold. The results are presented in the Statistical Analysis, Determinants of Heterogeneity, and Sensitivity Analysis sections, with additional findings provided in S7 and S9 Appendices.

iii. The methodological quality assessment of included studies must be described in detail, with studies categorized according to their quality scores (e.g., high, medium, and low quality). This classification should be transparently presented and consistently applied across all included articles.

Response: Thank you for this comment. We have expanded the description of the methodological quality assessment in the revised manuscript. Each included study was evaluated using the 12-item MEDQUARG checklist. The definition and scoring of each criterion are provided in S2 Appendix, while the completed item-level assessments and total scores for all included studies are presented in S3 Appendix. For the sensitivity analyses, studies were categorized as higher quality when they had a MEDQUARG score of ≥6 and lower quality when they had a score of <6. This classification was applied consistently across all included studies, and the results of the quality-restricted analyses are reported in S7 Appendix.

**Response to reviewers**

**Reviewer #1: Comments**
Reviewer #1: General comment: Authors must report database-specific keywords and search dates for all references. Absence of this information does not meet reporting standards and may warrant rejection. addressing this comments, the paper will be published

Response: We thank the reviewer for this important comment and agree that transparent reporting of the search strategy is essential. The full database-specific search strategy has been provided as S1 Appendix (Search Strategy), submitted with the manuscript. It reports, for each of the two searches (prevalence of substandard and falsified MNCH medicines and malaria). The individual search queries run in PubMed, Embase, and Web of Science, including all MeSH terms, free-text keywords, field tags (tiab / ab,ti,kw), and Boolean operators. The appendix also indicates the date each search was conducted (2 July 2025); and the total number of records retrieved per database. We have also added an explicit pointer to S1 Appendix in the Methods section (page 5) so that readers can locate the full strategy without ambiguity. We hope this fully addresses the reviewer’s concern.

Reviewer #2: Regarding question 2 about the statistical analysis, I am not a statistician so do not feel qualified to comment on this.

Response: We thank the reviewer for their transparency. No changes are required in response to this comment. We note that statistical analysis questions have been addressed in our responses to the other reviewer, and we remain happy to provide additional detail on the statistical methods if the editor would find it useful.

Overarching comments:

1. Overall, this is a well-written and clearly structured manuscript, with a strong discussion and thoughtful conclusions. I particularly appreciated the authors’ clear articulation of next steps, as analyses of this nature often generate important additional questions for future research.

Response: We thank the reviewer for these encouraging remarks, and for recognizing the value of the future research directions we have outlined. No changes are required in response to this comment.

2. The use of mystery-client purchasing as a factor in higher SF prevalence stands out to me. It would be helpful to briefly discuss why this could be associated with higher SF prevalence.

Response: Thank you for this insightful comment. We have expanded the Discussion to explain the observed association between mystery-client purchasing and higher SF prevalence. Mystery-client approaches may capture products as they are ordinarily sold because vendors are unaware that the purchase is part of a study, thereby reducing the possibility that vendors selectively provide higher-quality products to known investigators. These approaches may also be used more frequently in informal or otherwise higher-risk market settings. We have clarified that this association should not be interpreted as causal, because it may partly reflect differences in outlet type, sampling strategy, and the settings in which mystery-client methods were used.

3. The manuscript could also expand on the observation that many of the smaller studies were risk-based and discuss how this may have influenced the pooled results. For example, in Figure 3, corticosteroids appear to have the highest prevalence estimate, yet this is based on only two studies.

Response: Thank you for this important comment. We re-examined the study-level sampling data and revised our interpretation. Although funnel-plot asymmetry and Egger’s regression test indicated small-study effects, the dataset does not support attributing these effects primarily to targeted or risk-based sampling. Only a minority of the included studies used an explicitly targeted or risk-based approach, and neither of the two studies contributing corticosteroid data used this sampling method. The corticosteroid estimate was based on 32 samples from two studies: 22 samples from a convenience-sampled study and 10 samples from a randomly sampled survey. The study-specific failure proportions differed substantially, at 20/22 and 3/10, respectively. We have revised the Discussion to emphasize that the corticosteroid estimate is based on sparse and heterogeneous evidence and should not be interpreted as a representative continental prevalence estimate. We also describe the observed small-study effects more cautiously as potentially reflecting publication bias, selective reporting, and broader differences in therapeutic category, study setting, and methodological characteristics.

4. Also in Figure 3, it may be worth reconsidering whether antiprogesterone should be included, given that the sample size appears to be only 2, the prevalence estimate is 50%, and the confidence interval spans 0–100%. Unless I am misunderstanding the methodology, this also seems potentially inconsistent with the exclusion criterion of fewer than 10 collected samples. Clarification would be helpful.

Response: Thank you for identifying this issue. The exclusion criterion of fewer than 10 samples was applied at the study level rather than separately to each therapeutic category or country subgroup. The study contributing the antiprogestogen data included 22 eligible medicine samples collected across the included African countries and therefore met the study-level inclusion criterion; however, only two of those samples were antiprogestogens. We agree that an estimate based on two samples, with a confidence interval spanning approximately 0%–100%, is too imprecise for meaningful comparison with the other therapeutic categories. We have therefore removed the antiprogestogen estimate from Figure 3 and from the principal therapeutic-category comparisons in the Results. The underlying observations remain available in the study-level data appendices for completeness and transparency. We have also clarified in the Methods that the minimum sample-size criterion was applied at the study level. We also added a cautionary statement to the Discussion noting that several therapeutic-category subgroup estimates were based on very small numbers of studies and samples and should therefore be interpreted cautiously because of their limited precision and generalizability.

5. The very high prevalence of SF dexamethasone reported in Nigeria (95%) also appears noteworthy. It would be useful to indicate whether this estimate was driven by a risk-based study targeting products already suspected of quality concerns, as this would affect interpretation. If this was not the case, that would likewise be important to note.

Response: Thank you for raising this important point. We confirmed that the Nigerian dexamethasone estimate reported by Gabel et al. (2024) was based on convenience sampling and was not derived from an explicitly targeted or risk-based investigation. We have clarified this in the revised manuscript. The sampling method for this and all other included studies is also reported in S11 Appendix. Nevertheless, because convenience sampling is a non-probability approach and the samples were obtained from private and informal-sector outlets, the estimate may not be nationally representative and should be interpreted cautiously.

6. If not already provided elsewhere, it would be helpful to include a list of the specific medicines captured within each therapeutic category. For instance, it would be useful to clarify whether oxytocin was the only uterotonic included.

Response: Thank you for this helpful suggestion. The specific medicines included within each therapeutic category are listed in S11 Appendix, in the “Medicine” column alongside the corresponding therapeutic category. For example, the uterotonic category included oxytocin, misoprostol, and ergometrine; therefore, oxytocin was not the only uterotonic included. We have added a cross-reference to S11 Appendix in the revised manuscript.

7. In the section on geographic differences in prevalence, if the data are available, it would be informative to include a benchmark estimate from a high-income country or region to provide additional context for interpretation.

Response: Thank you for this helpful suggestion. We agree that a benchmark from a high-income country or region could provide useful context. However, we were unable to identify a sufficiently comparable and reliable estimate based on similar therapeutic categories, sampling frames, analytical methods, definitions, and study periods. We therefore did not include a benchmark.

8. The manuscript may also benefit from additional discussion of the relatively limited prevalence of falsified products compared with substandard products.

Response: Thank you for this important observation. We have added a brief discussion clarifying that the lower pooled prevalence of falsified products should not be interpreted as evidence that falsified medicines are absent or necessarily uncommon. Many included studies focused primarily on API content or other pharmacopeial quality parameters and did not consistently distinguish or were not able to distinguish falsified products from substandard products. In addition, samples described only as “poor quality” were classified as substandard unless the original investigators explicitly identified them as counterfeit or falsified. In addition, confirmed falsified products are often intercepted by law enforcement and customs, and may not reach the sampling frames of the studies pooled here. The pooled falsified estimate may therefore be conservative and partly reflect differences in testing and reporting practices across studies.

9. The phrase “threatening progress toward maternal and child mortality targets” could be strengthened to match the language in the discussion section: “the high prevalence of SF uterotonics likely contribute directly to preventable maternal mortality while failures in corticosteroid quality threaten neonatal survival among preterm infants. These findings collectively indicate that medicine quality is a major and under-recognized barrier to achieving regional targets for maternal and neonatal mortality reduction.”

Response: Thank you for this helpful suggestion. We have strengthened the conclusion of the Abstract to more clearly reflect the implications discussed in the manuscript while retaining appropriately cautious language because the review did not directly quantify mortality attributable to SF medicines.
