# Supplementary material for: Nearly one in five essential medicines fail quality standards in Africa: A systematic review and meta-analysis of MNCH medicines and antimalarials
Source: PLOS Glob Public Health. 2026 Sep 2;6(9):e0006326. doi: 10.1371/journal.pgph.0006326 (PMC13537570; doi:10.1371/journal.pgph.0006326)
Supplement: Peer review history

# Peer review: Decision Letter 0

**DOI:** 10.1371/journal.pgph.0006326.r001

**Authors:** Hannah Tappis

9 Jun 2026

PGPH-D-26-01235

Nearly One in Five Essential Medicines Fail Quality Standards in Africa: A Systematic Review and Meta-Analysis of MNCH Medicines and Antimalarials

PLOS Global Public Health

Dear Dr. Rusatira,

Thank you for submitting your manuscript to PLOS Global Public Health. Two reviewers have provided constructive feedback. After careful consideration, we feel that the manuscript has merit but does not fully meet PLOS Global Public Health’s publication criteria as it currently stands. Therefore, we invite you to submit a revised version of the manuscript that addresses the points raised during the review process, including confirmation of alignment with relevant PRISMA reporting standards available on https://www.equator-network.org/

Please submit your revised manuscript by Aug 08 2026 11:59PM. If you will need more time than this to complete your revisions, please reply to this message or contact the journal office at globalpubhealth@plos.org. When you're ready to submit your revision, log on to https://www.editorialmanager.com/pgph/ and select the 'Submissions Needing Revision' folder to locate your manuscript file.

Please include the following items when submitting your revised manuscript:

- A letter that responds to each point raised by the editor and reviewer(s). You should upload this letter as a separate file labeled 'Response to Reviewers'.
- A marked-up copy of your manuscript that highlights changes made to the original version. You should upload this as a separate file labeled 'Revised Manuscript with Track Changes'.
- An unmarked version of your revised paper without tracked changes. You should upload this as a separate file labeled 'Manuscript'.

Guidelines for resubmitting your figure files are available below the reviewer comments at the end of this letter.

As the corresponding author, your ORCID iD is verified in the submission system and will appear in the published article. PLOS supports the use of ORCID, and we encourage all coauthors to register for an ORCID iD and use it as well. Please encourage your coauthors to verify their ORCID iD within the submission system before final acceptance, as unverified ORCID iDs will not appear in the published article. *Only*  the individual author can complete the verification step; PLOS staff *cannot*  verify ORCID iDs on behalf of authors.

We look forward to receiving your revised manuscript.

Kind regards,

Hannah Tappis, DrPH, MPH

Academic Editor

PLOS Global Public Health

Journal Requirements:

1. We have noticed that you have uploaded Supporting Information files, but you have not included a list of legends. Please add a full list of legends for your Supporting Information files after the references list.

2. As required by our policy on Data Availability, please ensure your manuscript or supplementary information includes the following:

A numbered table of all studies identified in the literature search, including those that were excluded from the analyses.

For every excluded study, the table should list the reason(s) for exclusion.

If any of the included studies are unpublished, include a link (URL) to the primary source or detailed information about how the content can be accessed.

A table of all data extracted from the primary research sources for the systematic review and/or meta-analysis. The table must include the following information for each study:

Name of data extractors and date of data extraction

Confirmation that the study was eligible to be included in the review.

All data extracted from each study for the reported systematic review and/or meta-analysis that would be needed to replicate your analyses.

If data or supporting information were obtained from another source (e.g. correspondence with the author of the original research article), please provide the source of data and dates on which the data/information were obtained by your research group.

If applicable for your analysis, a table showing the completed risk of bias and quality/certainty assessments for each study or outcome.  Please ensure this is provided for each domain or parameter assessed. For example, if you used the Cochrane risk-of-bias tool for randomized trials, provide answers to each of the signalling questions for each study. If you used GRADE to assess certainty of evidence, provide judgements about each of the quality of evidence factor. This should be provided for each outcome.

An explanation of how missing data were handled.

This information can be included in the main text, supplementary information, or relevant data repository. Please note that providing these underlying data is a requirement for publication in this journal, and if these data are not provided your manuscript might be rejected.

3. We note that your Data Availability Statement is currently as follows: "All data underlying this systematic review and meta-analysis are available within the manuscript and its supplementary appendices, including the full search strategies (S1 Appendix), MEDQUARG quality assessment scores (S3 Appendix), and study-level prevalence estimates (S5 Appendix). The dataset used for meta-analysis, including extracted study-level data, effect sizes, and meta-regression variables, will be made available upon reasonable request to the Johns Hopkins BESAFE program at besafe@jh.edu."

Please confirm at this time whether or not your submission contains all raw data required to replicate the results of your study. Authors must share the “minimal data set” for their submission. PLOS defines the minimal data set to consist of the data required to replicate all study findings reported in the article, as well as related metadata and methods (https://journals.plos.org/plosone/s/data-availability#loc-minimal-data-set-definition).

For example, authors should submit the following data:

- The values behind the means, standard deviations and other measures reported;

- The values used to build graphs;

- The points extracted from images for analysis.

Authors do not need to submit their entire data set if only a portion of the data was used in the reported study.

If your submission does not contain these data, please either upload them as Supporting Information files or deposit them to a stable, public repository and provide us with the relevant URLs, DOIs, or accession numbers. For a list of recommended repositories, please see https://journals.plos.org/plosone/s/recommended-repositories.

If there are ethical or legal restrictions on sharing a de-identified data set, please explain them in detail (e.g., data contain potentially sensitive information, data are owned by a third-party organization, etc.) and who has imposed them (e.g., an ethics committee). Please also provide contact information for a data access committee, ethics committee, or other institutional body to which data requests may be sent. If data are owned by a third party, please indicate how others may request data access.

4. Some material included in your submission may be copyrighted. According to PLOS’s copyright policy, authors who use figures or other material (e.g., graphics, clipart, maps) from another author or copyright holder must demonstrate or obtain permission to publish this material under the Creative Commons Attribution 4.0 International (CC BY 4.0) License used by PLOS journals. Please closely review the details of PLOS’s copyright requirements here: PLOS Licenses and Copyright. If you need to request permissions from a copyright holder, you may use PLOS's Copyright Content Permission form.

Please respond directly to this email or email the journal office and provide any known details concerning your material's license terms and permissions required for reuse, even if you have not yet obtained copyright permissions or are unsure of your material's copyright compatibility.

Potential Copyright Issues:

Figure 2: please (a) provide a direct link to the base layer of the map (i.e., the country or region border shape) and ensure this is also included in the figure legend; and (b) provide a link to the terms of use / license information for the base layer image or shapefile. We cannot publish proprietary or copyrighted maps (e.g. Google Maps, Mapquest) and the terms of use for your map base layer must be compatible with our CC-BY 4.0 license.

Note: if you created the map in a software program like R or ArcGIS, please locate and indicate the source of the basemap shapefile onto which data has been plotted.

If your map was obtained from a copyrighted source please amend the figure so that the base map used is from an openly available source. Alternatively, please provide explicit written permission from the copyright holder granting you the right to publish the material under our CC-BY 4.0 license.

Please note that the following CC BY licenses are compatible with PLOS license: CC BY 4.0, CC BY 2.0 and CC BY 3.0, meanwhile such licenses as CC BY-ND 3.0 and others are not compatible due to additional restrictions.

If you are unsure whether you can use a map or not, please do reach out and we will be able to help you. The following websites are good examples of where you can source open access or public domain maps:

* U.S. Geological Survey (USGS) - All maps are in the public domain. (http://www.usgs.gov)

* PlaniGlobe - All maps are published under a Creative Commons license so please cite “PlaniGlobe, http://www.planiglobe.com, CC BY 2.0” in the image credit after the caption. (http://www.planiglobe.com/?lang=enl)

* Natural Earth - All maps are public domain. (http://www.naturalearthdata.com/about/terms-of-use/)

If the reviewer comments include a recommendation to cite specific previously published works, please review and evaluate these publications to determine whether they are relevant and should be cited. There is no requirement to cite these works unless the editor has indicated otherwise.

Additional Editor Comments (if provided):

The first reviewer provided this additional feedback: The information presented is critical for informing policy and strengthening the body of knowledge on SFMP prevention. However, the manuscript requires substantial review to align with international reporting guidelines. In particular, issues related to publication bias should be explicitly addressed. Additionally, subgroup meta-analyses based on quality control parameters should be conducted and clearly reported. The methodological quality assessment of included studies must be described in detail, with studies categorized according to their quality scores (e.g., high, medium, and low quality). This classification should be transparently presented and consistently applied across all included articles.

[Note: HTML markup is below. Please do not edit.]

Reviewers' comments:

Reviewer's Responses to Questions

Comments to the Author

1. Does this manuscript meet PLOS Global Public Health’s publication criteria?

Reviewer #1: Yes

Reviewer #2: Yes

**********

2. Has the statistical analysis been performed appropriately and rigorously?

Reviewer #1: No

Reviewer #2: I don't know

**********

3. Have the authors made all data underlying the findings in their manuscript fully available (please refer to the Data Availability Statement at the start of the manuscript PDF file)?

The PLOS Data policy

Reviewer #1: Yes

Reviewer #2: Yes

**********

4. Is the manuscript presented in an intelligible fashion and written in standard English?

Reviewer #1: No

Reviewer #2: Yes

**********

Reviewer #1: General comment: Authors must report database-specific keywords and search dates for all references. Absence of this information does not meet reporting standards and may warrant rejection. addressing this comments, the paper will be published

Reviewer #2: Regarding question 2 about the statistical analysis, I am not a statistician so do not feel qualified to comment on this.

Overarching comments:

- Overall, this is a well-written and clearly structured manuscript, with a strong discussion and thoughtful conclusions. I particularly appreciated the authors’ clear articulation of next steps, as analyses of this nature often generate important additional questions for future research.

- The use of mystery-client purchasing as a factor in higher SF prevalence stands out to me. It would be helpful to briefly discuss why this could be associated with higher SF prevalence.

- The manuscript could also expand on the observation that many of the smaller studies were risk-based and discuss how this may have influenced the pooled results. For example, in Figure 3, corticosteroids appear to have the highest prevalence estimate, yet this is based on only two studies.

- Also in Figure 3, it may be worth reconsidering whether antiprogesterone should be included, given that the sample size appears to be only 2, the prevalence estimate is 50%, and the confidence interval spans 0–100%. Unless I am misunderstanding the methodology, this also seems potentially inconsistent with the exclusion criterion of fewer than 10 collected samples. Clarification would be helpful.

- The very high prevalence of SF dexamethasone reported in Nigeria (95%) also appears noteworthy. It would be useful to indicate whether this estimate was driven by a risk-based study targeting products already suspected of quality concerns, as this would affect interpretation. If this was not the case, that would likewise be important to note.

- If not already provided elsewhere, it would be helpful to include a list of the specific medicines captured within each therapeutic category. For instance, it would be useful to clarify whether oxytocin was the only uterotonic included.

- In the section on geographic differences in prevalence, if the data are available, it would be informative to include a benchmark estimate from a high-income country or region to provide additional context for interpretation.

- The manuscript may also benefit from additional discussion of the relatively limited prevalence of falsified products compared with substandard products.

- The phrase “threatening progress toward maternal and child mortality targets” could be strengthened to match the language in the discussion section: “the high prevalence of SF uterotonics likely contribute directly to preventable maternal mortality while failures in corticosteroid quality threaten neonatal survival among preterm infants. These findings collectively indicate that medicine quality is a major and under-recognized barrier to achieving regional targets for maternal and neonatal mortality reduction.”

**********

what does this mean?). If published, this will include your full peer review and any attached files.

**Do you want your identity to be public for this peer review?**  If you choose “no”, your identity will remain anonymous but your review may still be made public.

For information about this choice, including consent withdrawal, please see our Privacy Policy

Reviewer #1: **Yes:**  Yesuneh Tefera Mekasha

Reviewer #2: No

**********

[NOTE: If reviewer comments were submitted as an attachment file, they will be attached to this email and accessible via the submission site. Please log into your account, locate the manuscript record, and check for the action link "View Attachments". If this link does not appear, there are no attachment files.]

# Author comment: Author response to Decision Letter 1

**DOI:** 10.1371/journal.pgph.0006326.r002

22 Jul 2026

Attachment Submitted filename: Response to Reviewers.docx

# Editor report: Decision Letter 1

**DOI:** 10.1371/journal.pgph.0006326.r003

**Authors:** Hannah Tappis

24 Jul 2026

Nearly One in Five Essential Medicines Fail Quality Standards in Africa: A Systematic Review and Meta-Analysis of MNCH Medicines and Antimalarials

PGPH-D-26-01235R1

Dear Rusatira,

We are pleased to inform you that your manuscript 'Nearly One in Five Essential Medicines Fail Quality Standards in Africa: A Systematic Review and Meta-Analysis of MNCH Medicines and Antimalarials' has been provisionally accepted for publication in PLOS Global Public Health.

Before your manuscript can be formally accepted you will need to complete some formatting changes, which you will receive in a follow up email. A member of our team will be in touch with a set of requests.

Please note that your manuscript will not be scheduled for publication until you have made the required changes, so a swift response is appreciated.

IMPORTANT: The editorial review process is now complete. PLOS will only permit corrections to spelling, formatting or significant scientific errors from this point onwards. Requests for major changes, or any which affect the scientific understanding of your work, will cause delays to the publication date of your manuscript.

If your institution or institutions have a press office, please notify them about your upcoming paper to help maximize its impact. If they'll be preparing press materials, please inform our press team as soon as possible -- no later than 48 hours after receiving the formal acceptance. Your manuscript will remain under strict press embargo until 2 pm Eastern Time on the date of publication. For more information, please contact globalpubhealth@plos.org.

Thank you again for supporting Open Access publishing; we are looking forward to publishing your work in PLOS Global Public Health.

Best regards,

Hannah Tappis, DrPH, MPH

Academic Editor

PLOS Global Public Health

***********************************************************

Reviewer Comments (if any, and for reference):
